# Evidence That *PbrSAUR72* Contributes to Iron Deficiency Tolerance in Pears by Facilitating Iron Absorption

**DOI:** 10.3390/plants12112173

**Published:** 2023-05-30

**Authors:** Guoling Guo, Tao Yu, Haiyan Zhang, Meng Chen, Weiyu Dong, Shuqin Zhang, Xiaomei Tang, Lun Liu, Wei Heng, Liwu Zhu, Bing Jia

**Affiliations:** 1 State Key Laboratory of Fruit Biology, School of Horticulture, Anhui Agricultural University, Hefei 230036, China; guolingguo@stu.ahau.edu.cn (G.G.); m18851117598@163.com (T.Y.);; 2 Agricultural Experimental Center of Guiyang, Guiyang Agriculture and Rural Bureau, Guiyang 550018, China; 3 Singleron Biotechnology Co., Ltd., Nanjing 210000, China

**Keywords:** pear, indoleacetic acid, *PbrSAUR72*, Fe deficiency tolerance, reactive oxygen species

## Abstract

Iron is an essential trace element for plants; however, low bioactive Fe in soil continuously places plants in an Fe-deficient environment, triggering oxidative damage. To cope with this, plants make a series of alterations to increase Fe acquisition; however, this regulatory network needs further investigation. In this study, we found notably decreased indoleacetic acid (IAA) content in chlorotic pear (*Pyrus bretschneideri* Rehd.) leaves caused by Fe deficiency. Furthermore, IAA treatment slightly induced regreening by increasing chlorophyll synthesis and Fe^2+^ accumulation. At that point, we identified *PbrSAUR72* as a key negative effector output of auxin signaling and established its close relationship to Fe deficiency. Furthermore, the transient *PbrSAUR72* overexpression could form regreening spots with increased IAA and Fe^2+^ content in chlorotic pear leaves, whereas its transient silencing does the opposite in normal pear leaves. In addition, cytoplasm-localized PbrSAUR72 exhibits root expression preferences and displays high homology to *AtSAUR40*/*72*. This promotes salt tolerance in plants, indicating a putative role for *PbrSAUR72* in abiotic stress responses. Indeed, transgenic plants of *Solanum lycopersicum* and *Arabidopsis thaliana* overexpressing *PbrSAUR72* displayed less sensitivity to Fe deficiency, accompanied by substantially elevated expression of Fe-induced genes, such as *FER*/*FIT*, *HA*, and *bHLH39*/*100*. These result in higher ferric chelate reductase and root pH acidification activities, thereby hastening Fe absorption in transgenic plants under an Fe-deficient condition. Moreover, the ectopic overexpression of *PbrSAUR72* inhibited reactive oxygen species production in response to Fe deficiency. These findings contribute to a new understanding of *PbrSAURs* and its involvement in Fe deficiency, providing new insights for the further study of the regulatory mechanisms underlying the Fe deficiency response.

## 1. Introduction

Iron (Fe) is an indispensable element for plant growth and development. It has functions in many critical processes throughout life, including respiration, photosynthesis, oxidative stress responses, and pathogen defense [1]. However, because of the low bioavailability of Fe in most areas with alkaline or calcified soils, the potential for plants to access this essential element can often be very limited, causing severe chlorosis in leaves and shoots [2,3]. This has become a common nutrition disorder in agriculture [4], reducing the yield and quality of plants.

China is the largest producer of pear (*Pyrus* spp.) globally, and the cultivation area and yield are 0.98 million hectares and 18.98 million tons, respectively (https://www.fao.org/faostat/en/#data/QCL, accessed on 10 February 2023). Hence, pear production has an important role in the fruit industry in China. Nonetheless, its growth, development, and productivity are frequently hampered by Fe deficiency [5]. To address this, fruit growers have taken various physical and chemical measures which have resulted in some negative side effects, such as environmental harm and reduced food safety. A time-saving and effective way to combat Fe deficiency is to breed Fe deficiency-tolerant varieties via genetic and transgenic approaches. Therefore, understanding the molecular mechanisms of Fe absorption and transport is fundamental to achieving strong Fe deprivation tolerance in breeding projects. Subsequently, it could be of great significance for expanding the distribution of pear trees into areas with alkaline or calcified soils.

Over the past few decades, Fe deficiency responses (FDRs) have been intensively studied in *Arabidopsis* [6], soybean [7], rice [8], apple [9], and tomato [10]. When challenged with Fe deficiency, plants employ two main mechanisms for Fe acquisition from the soil. They can be grouped into reduction mechanism I, used in dicots and non-graminaceous monocots, and mechanism II, where Fe is chelated, which occurs only in graminaceous plants [11,12]. Like *Arabidopsis*, pear uses mechanism I to take up Fe, where a proton efflux acts to acidify the rhizosphere via the action of H^+^-ATpase 2 (AHA2) [13], allowing ferric reductase oxidase 2 (FRO2) to reduce insoluble Fe^3+^ into absorbable Fe^2+^ [14]. Then, Fe^2+^ is subsequently imported from the soil into the root cell through iron-regulated transporter 1 (IRT1) for Fe utilization [15,16]. All of these genes are induced under Fe-deficient conditions. They are regulated by several transcription factors, particularly those of the basic helix–loop–helix (bHLH) family [17,18,19], which are widely distributed in plants and highly conserved in the regulation of various abiotic stresses [20].

Although the molecular mechanisms underlying Fe deficiency resistance are exclusive and complex, mounting evidence suggests that auxin is a key conduit for FDR. Fe deficiency can lead to increased indoleacetic acid (IAA) content in the shoot apex of *Malus*. IAA, in turn, could mimic FDR via the induction of Fe(III) reductase activity and proton extrusion [21]. In addition, blocked polar auxin transport cannot activate Fe stress responses at the transcriptional or functional levels in cucumber [22]. In line with its facilitative role in improving Fe deficiency tolerance, the exogenous application of gamma-aminobutyric acid (GABA) could enhance the ability to amplify Fe metabolic processes in auxin signaling by elevating the expression of YUCCA4 (YUC4) and PIN-formed1 (PIN1), which are related to auxin biosynthesis and transport, whereas 1-naphthylphthalamic acid, an auxin transport inhibitor, eliminates GABA-mediated changes [23]. Moreover, IAA can interact with nitric oxide to regulate root morphogenesis under stress due to Fe deficiency, thereby responding to abiotic stress [24]. Therefore, IAA plays a positive role in ameliorating the sensitivity of plants to Fe deficiency. 

*Small auxin upregulated RNA* (*SAUR*) genes belong to one of the IAA signaling pathway downstream responsive genes known to be involved in growth, development, and stress adaptation. In *Arabidopsis*, *AtSAUR10*/*36*/*39*/*49* controls leaf senescence by mediating senescence-associated gene (*AtSAG201*) expression [25,26,27,28]. *AtSAUR26* has been reported to play an active role in adapting to the climate changes of *Arabidopsis* [29]. In addition, *AtSAUR41* overexpression could enhance the tolerance of *Arabidopsis* seedlings to salinity stress [30], suggesting that *SAURs* are of great importance for plants’ survival in adverse environments.

Several *SAUR* gene functions have been studied in plants; however, the mechanism of FDR in pears has yet to be elucidated. In this study, we revealed that IAA is a crucial mediator in FDR in pear plants. We identified *PbrSAUR72* as a positive regulator of FDR in an IAA signaling-independent mode, the transient overexpression of which formed regreening spots in the Fe-deficient chlorotic leaves of pear, where the Fe^2+^ content and IAA concentration were both increased. Notably, we found that ectopic *PbrSAUR72* overexpression enhanced tolerance to Fe deficiency stress and led to higher Fe accumulation in both tomato (*Solanum lycopersicum*) and *A. thaliana* by inhibiting reactive oxygen species (ROS) production and upregulating the expression of Fe-related genes, such as *FER*/*FIT*, *IRT*, *FRO*, *HA*, and *bHLH39*/*100*. Overall, our results broaden the understanding of the function of *SAUR* genes in mediating Fe deficiency tolerance and provide new insights into the regulatory mechanisms underlying this process.

## 2. Results

### 2.1. Exogenous IAA Treatment Slightly Induces Re-Greening of Fe-Deficienty Chlorotic Pear Leaves 

**Figure 1:**
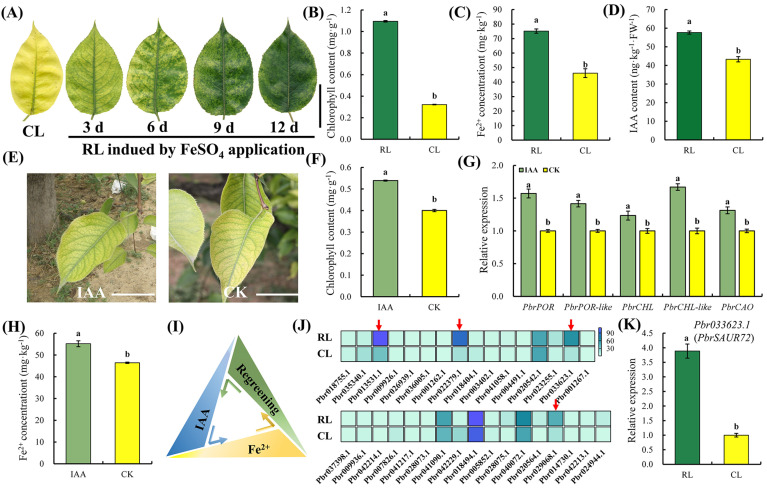
The effect of indoleacetic acid (IAA) on Fe deficiency responses and identification of *PbrSAUR* genes in response to Fe deficiency stress in pear. (**A**) Fe-deficient chlorotic leaf (CL) and 0.2% FeSO_4_-induced regreening leaf (RL) at 3, 6, 9, and 12 d after treatments of ‘Dangshansuli’ (*Pyrus bretschneideri* Rehd.) grafted on ‘Duli’ (*Pyrus betulifolia* Bge.) rootstock in the field, respectively. Bars = 5 cm. (**B**) Chlorophyll content, (**C**) Fe^2+^ concentration, and (**D**) IAA abundance in RL and CL of ‘Dangshansuli’ after 9 d of treatment. (**E**) Exogenous IAA treatment induced the regreening of CL. To study the function of auxin on Fe deficiency responses in pear, 10 μM IAA was sprayed evenly onto CL, with distilled water used as the control group. Bars = 5 cm. (**F**) Analysis of total chlorophyll content and (**G**) the expression of chlorophyll synthesis-related genes in CL with or without IAA treatment. The expression level detected in CL without IAA application was used as the reference and set to ‘1’. (**H**) Determination of Fe^2+^ content in IAA-treated and distilled water-treated CL. (**I**) Schematic diagram of the relationship between IAA content, Fe^2+^ content, and the re-greening phenomenon of CL constructed from previous studies and this study. (**J**) Thirty-three genes expressed in both RL and CL based on the previously established RNA sequencing; the red arrows indicate statistical significances in the expression of genes between RL and CL. (**K**) qRT-PCR analysis of *PbrSAUR72* in RL and CL. The expression level detected in CL was used as the reference and set to ‘1’. *PbrGAPDH* was used as the internal control. Values shown are the mean ± SE (*n* = 3) of three biological replicates. Student’s *t*-test: Different lowercase letters above the columns indicate significant differences at *p* < 0.05.

Exogenous 0.2% FeSO_4_ solution has previously been demonstrated to promote leaf FDR by regulating IAA synthesis, resulting in the regreening of chlorotic leaves (CL) [31]. We sprayed CL with 0.2% FeSO_4_ solution to verify this result, and recorded its changes at 3, 6, 9, and 12 d after treatment. The results showed that FeSO_4_ treatment induced the formation of mottled greening spots on CL, which were so-called regreening leaves (RL). Notably, this phenomenon became more pronounced with time in the following two weeks (Figure 1A). However, the mottled green spots on CL gradually disappeared after that time. Thus, we collected the leaf samples at 6 d after treatment to explore the changes in the Fe^2+^ and IAA contents. Consistently with the phenomenon observed, the chlorophyll content was significantly increased in RL compared to that in CL (Figure 1B). In line with the previous results, the level of Fe^2+^ and IAA in RL were also prominently elevated in RL compared with CL (Figure 1C,D). Therefore, IAA may be associated with Fe-deficient chlorosis in pears.

The effect of IAA on FDR in pear trees was investigated by spraying IAA with different doses evenly on CL. After 14 days of treatment, mottled green spots did not appear on the leaf surface of CL in all treatments; however, slight green around the veins appeared in CL with 10 μM IAA application (Figure 1E and Figure S1). Moreover, the expression of genes related to chlorophyll synthesis notably increased after 10 μM IAA application (Figure 1G), leading to a markedly higher chlorophyll content (Figure 1F). Further, a significant increase in the Fe^2+^ content of the samples around the veins was also observed (Figure 1H). These results suggest that IAA can potentially convert CL back to green by mediating Fe metabolism. However, likely because of its interaction with various phytohormones and the unavoidable effect of the environment, we failed to observe the regreening of CL. Nonetheless, combined with the previous results, we were able to summarize the connection between IAA, Fe^2+^, and FDR (Figure 1I). Therefore, we concluded that IAA might positively regulate FDR in pear trees.

### 2.2. Identification of the Pear Fe Deficiency-Responsive Gene PbrSAUR72

Auxin signaling is imperative for auxin function. Considering the crucial physiological functions of *SAUR*, a key effector of auxin-responsive downstream functional genes in auxin signaling, which are in control of factors such as leaf senescence and stress responses [29,30], we speculated that *PbrSAUR* family genes might also be instrumental in FDR. To identify the *PbrSAUR* genes involved in FDR, we analyzed the previously established transcriptome of RL and CL for ‘Dangshansuli’. Among the differentially expressed genes, 85 *PbrSAUR* genes were identified in RL and CL, and only 33 *PbrSAUR* genes were detected in both samples (Figure 1J; Table S2). Moreover, we observed that only four genes, *Pbr033623.1*, *Pbr022379.1*, *Pbr013531.1*, and *Pbr029068.1*, were significantly more highly expressed in RL than in CL (Figure 1J; Table S2). Additionally, most *PbrSAURs* were expressed in the RL (Figure 1J; Table S2). Then, we verified the RNA sequencing results via real-time quantitative reverse transcription polymerase chain reaction (qRT-PCR) to determine the expression of these four genes in the RL and CL of pear plants. The results were consistent with the transcriptome analysis (Figure 1K and Figure S2A–C). Consequently, the higher IAA content and *PbrSAURs* expression in RL led us to label these as positive mediators of FDR in the IAA signaling pathway. We expect that the overexpression phenotype would show less sensitivity to Fe deficiency.

**Figure 2:**
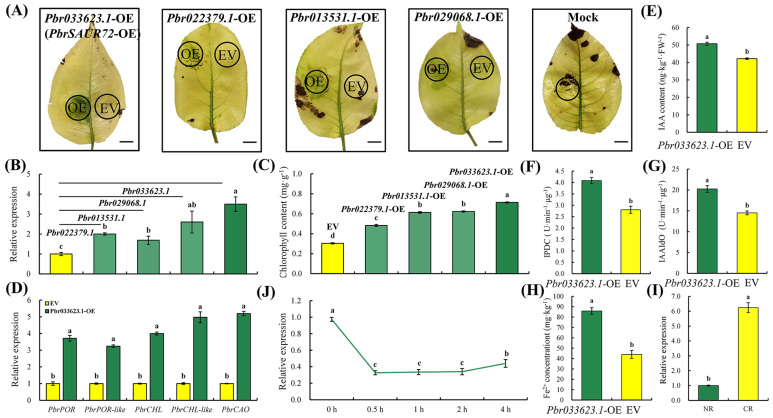
Transient *PbrSAUR* overexpression alleviated leaf chlorosis and promotes Fe^2+^ content and indoleacetic acid (IAA) accumulation in chlorotic pear leaves (*Pyrus bretschneideri* Rehd.). (**A**) Four genes highly expressed in the regreening leaf (RL), including *Pbr033623.1* (*PbrSAUR72*), *Pbr022379.1*, *Pbr013531.1*, and *Pbr029068.1*, were overexpressed in the CL via syringe injection. An injection with empty vector (EV) or no treatment to the symmetrical leaf surface of the same leaf was used as the control and mock conditions, respectively. The phenotypes were observed at 14 d after injection. Bars = 1 cm. Meanwhile, (**B**) the corresponding gene expression and (**C**) the chlorophyll content in the injection site were measured to show the changes in the leaf color. (**D**) Expression analysis of chlorophyll synthesis-associated genes in control and in the re-greening spot with overexpressing gene, *Pbr033623.1*. (**E**) Analysis of activities for IAA synthesis-dependent IPDC enzyme and (**F**) IAAIdO enzyme as well as (**G**) IAA content and (**H**) Fe^2+^ content in the regreening spot with *Pbr033623.1* overexpression. Injection into the symmetrical leaf surface of the same leaf with empty vector (EV) was used as the control condition. (**I**,**J**) *PbrSAUR72* positively and negatively responded to Fe deficiency and IAA treatment, respectively. (**I**) *Pbr033623.1* (*PbrSAUR72*) expression in the roots of pear trees in both normal (NR) and Fe-deficient conditions (CR) was determined via qRT-PCR. The expression level detected in NR was used as the reference and set to ‘1’. (**J**) *PbrSAUR72* negatively responded to IAA treatment. To determine the responses of *PbrSAUR72* to IAA, 10 μM IAA was sprayed on normal leaves (NL) of pear plants (*P. bretschneideri* Rehd.), and *PbrSAUR72* expression was analyzed by qRT-PCR at 0.5 h, 1 h, 2 h, and 4 h after IAA treatment, respectively. The expression level detected in NL before IAA treatment was used as the reference and set to ‘1’. *PbrGAPDH* was used as the internal control. Values shown are the mean ± SE (*n* = 3) of three biological replicates. Student’s *t*-test: Different lowercase letters above the columns indicate significant differences at *p* < 0.05.

To confirm this hypothesis, we transiently introduced the overexpression fusion vectors into CL of ‘Dangshansuli’ via a sterile injector. We expected the CL to show a regreening phenotype similar to that of the RL induced by FeSO_4_. Consistently, when transiently overexpressing *Pbr033623.1*, *Pbr022379.1*, *Pbr013531.1*, and *Pbr029068.1* in CL, respectively (Figure 2B), the area infiltrated with *PbrSAUR* genes turned green in a mottled pattern accompanied by notably higher chlorophyll content, especially *Pbr033623.1*. In contrast, spots injected with an empty vector remained chlorotic, with lower chlorophyll content at 14 d after treatment (Figure 2A,C). Notably, the expression of chlorophyll synthesis-related genes in the regreening spots (at 6 d after treatment) infiltrated with *Pbr033623.1* genes were markedly upregulated (Figure 2D), which can partly explain their increased chlorophyll content (Figure 2C). In addition, transient *Pbr033623.1* overexpression enhanced the enzyme activities of 3-indolepyruvate decarboxylase (IPDC) and the indolealdehyde oxidase (IAAIdO) (Figure 2F,G), significantly increasing the IAA content (Figure 2E). Moreover, as a key element for regreening, the Fe^2+^ content was significantly increased in the *PbrSAUR72*-injected spots (Figure 2H). This meant that the *PbrSAURs* ameliorated the Fe-deficient chlorosis of the pear, meeting our expectations. Given the critical function of the root system in FDR, we analyzed the expression of related genes in the roots of normal (NR) and Fe-deficient pear trees (CR). The expression of these four genes was significantly upregulated in the roots under Fe-deficient conditions (Figure 2I and Figure S2D–F), indicating that Fe deficiency significantly induced the expression of *PbrSAURs*. Hence, *PbrSAURs* (*Pbr033623.1*, *Pbr022379.1*, *Pbr013531.1*, and *Pbr029068.1*) positively mediated FDR in pears.

As seen in Figure 2, the relative expressions of *Pbr033623.1* and *Pbr029068.1* (Figure 2B) were higher and statistically comparable; however, only the soil plant analysis development (SPAD) value of *Pbr033623.1*-OE (Figure 2C) was higher than that of the others according to the statistical significance. These results suggest that the regreening degree of CL mainly depends on the kind of gene, but not enhanced expression. To illustrate the above point, we chose *Pbr022379.1* and *Pbr033623.1* to repeat the experiment and designed two different schemes with volume ratios of 1:1 and 2:1 (*Pbr022379.1*/*Pbr033623.1*). Although the regreening degree of *Pbr022379.1*-OE was slightly enhanced with the doubling of the injection volume, the regreening degree of *Pbr033623.1*-OE was consistently greater than that of the *Pbr022379.1*-OE (Figure S2G). This confirms the above results. Hence, the gene’s function dominated the regreening degree to a great extent. Moreover, since the effect of *Pbr033623.1* was the strongest among the four genes, we selected *Pbr033623.1* for further study.

### 2.3. Sequence Analysis, Subcellular Localization, Phylogenetic Analysis, and Expression Pattern of PbrSAUR72

**Figure 3:**
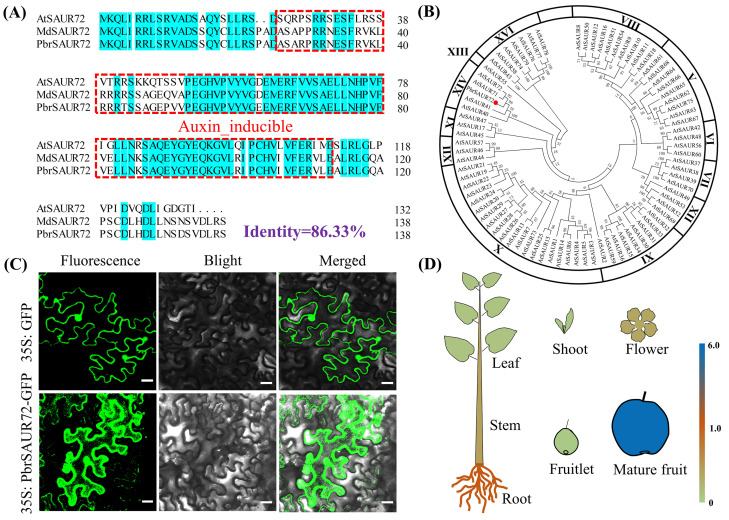
Sequence analysis, phylogenetic tree analysis, subcellular localization, and expression patterns the PbrSAUR72 protein. (**A**) Multiple sequence alignments of PbrSAUR72 and its homologous sequence from *Malus domestica* and *Arabidopsis thaliana*. The sequences used for comparison with *Pyrus bretschneideri* PbrSAUR72 (Pbr033623.1) are *Malus domestica* MdSAUR72 (accession: XM_008384868) and *Arabidopsis thaliana* AtSAUR72 (accession: AT3G12830). The same amino acids are displayed against a light blue background. The sequences’ similarity is marked in bold purple font. The red frame indicates the auxin-inducible structure. (**B**) Phylogenetic tree analyses of PbrSAUR72 in *P. bretschneideri* and *AtSAUR* genes in *A. thaliana* constructed from amino acid multiple sequence alignments using the MEGA program (Version 7.0). The optimal tree, with a sum of branch length = 14.91850183, is shown. The number next to each branch indicates the bootstrapped value based on 1000 runs. Frames were used to distinguish the groups or subgroups of the SAURs. The PbrSAUR72 in *P. bretschneideri* is denoted by red dots. (**C**) Subcellular localization of the PbrSAUR72 protein. Tobacco epidermal cells were transiently transformed with constructs containing either the control (35S: GFP, GFP alone) or fusion plasmid (35S: *PbrAUR72*-GFP). Images under fluorescence (left) and blight field (middle), as well as the merged images (right), are shown on the right; scale bar = 25 µm. (**D**) Heat map of tissue-specific *PbrSAUR72* expression in roots, leaves, stems, flowers, fruitlets, and mature fruit at 175 days after full bloom of pear plants (*P. bretschneideri* Rhed.). This gene’s expression level in the roots served as the reference and was set to ‘1’. *PbrGAPDH* was used as the internal control. Values shown are the mean ± SE (*n* = 3) of three biological replicates.

*Pbr033623.1* and its homology in *Malus domestica* are annotated as the *PbrSAUR71*-like and *MdSAUR72* genes in the genomes of these two plant species in the National Center for Biotechnology Information (NCBI). This allowed us to refer to *Pbr033623.1* as *PbrSAUR72* hereafter in this study. The results showed that the transcript of *PbrSAUR72* was arrested quickly by IAA, and at a lower level with prolonged IAA treatment (Figure 2J), indicating that *PbrSAUR72*-mediated FDR in pears is independent of IAA signaling. The full-length coding sequence of *PbrSAUR72* is 420 bp long. It encodes a protein of approximately 15.85 KD (140 amino acids) with an auxin-inducible domain at its central terminus (Figure 3A), which is highly conserved and essential for SAUR functions. Phylogenetic analysis showed that *PbrSAUR72* belongs to the SAUR gene family and is closely related to *AtSAUR72*/*71*/*41*/*40* in *A. thaliana* (Figure 3B). Green fluorescent protein (GFP) was fused to the C-terminus of PbrSAUR72 to examine subcellular localization and transient expression in tobacco leaves. Microscopic visualization of PbrSAUR72 indicated that it had cytoplasm localization preference (Figure 3C), suggesting that PbrSAUR72 possibly plays a role in cytoplasmic physiological metabolism.

Total RNA was extracted from the roots, stems, leaves, flowers, shoots, fruitlets (15 days after full bloom), and mature fruits (175 days after full bloom) of the pear (*P. bretschneideri* Rehd.) to study the tissue-specific expression pattern of *PbrSAUR72*. The qRT-PCR assay showed that *PbrSAUR72* was expressed in all tested tissues. However, expression was very low in vegetative tissues, such as leaves and fruitlets. Notably, it was highly expressed in roots and peaked in mature fruits (Figure 3D), indicating that *PbrSAUR72* may act as a regulator of root growth, fruit development, and ripening. To further understand the function of *PbrSAUR72*, we constructed a silencing vector and analyzed its function in the NL (Figure S3B). The results revealed that silencing *PbrSAUR72* inhibited chlorophyll synthesis (Figure S3D) and accelerated chlorophyll degradation (Figure S3E), leading to chlorosis (Figure S3A,C). In addition, the loss of function of *PbrSAUR72* significantly reduced the Fe^2+^ content in the chlorotic spots (Figure S3F). These findings are consistent with the positive role of *PbrSAUR72* in FDR in pear.

### 2.4. Heterologous Expression of PbrSAUR72 Confers Strong Tolerance to Fe Deficiency of Both Solanum lycopersicum and A. thaliana

**Figure 4:**
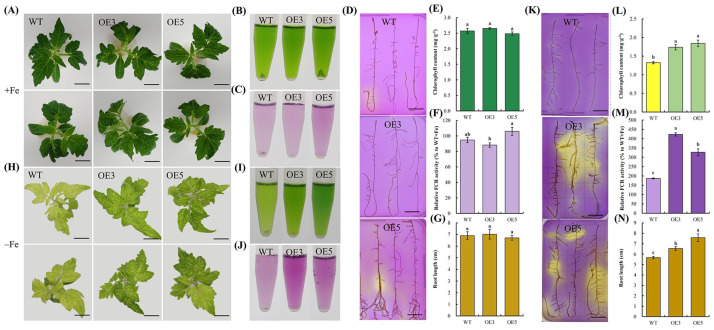
*PbrSAUR72*-overexpressing transgenic tomato plants showed higher tolerances to Fe deficiency. (**A**–**G**) No significant differences were observed between transgenic and wild-type (WT) tomato plants under Fe sufficiency conditions. (**A**) Phenotypes of transgenic tomato plants and WT tomato plants under Fe-sufficient conditions. The variations in the leaf colors of seedlings of WT and transgenic lines 3 (OE3) and 5 (OE5), cultured on MS medium for about three weeks, were recorded. (**B**) Total chlorophyll accumulation (visualization of 0.2 g of plant leaves soaked in extraction solution) and (**C**) WT, OE3, and OE5 content in leaves under Fe-sufficient conditions. (**D**) Visualization and (**E**) determination of root ferric chelate reductase (FCR) activity in OE3 and OE5 compared to that in WT under Fe-sufficient conditions. (**F**) The localization of root acidification activity and (**G**) root length analysis in WT, OE3, and OE5 under Fe-sufficient conditions. (**H**–**N**) *PbrSAUR72* overexpression enhanced the Fe deficiency responses in tomato under Fe-sufficient conditions. (**H**) Phenotypes of transgenic and WT tomato plants under Fe-deficient conditions. Seedlings of WT and transgenic lines 3 (OE3) and 5 (OE5) with primary roots approximately 2–3 cm long, cultured on MS medium, were transferred to –Fe medium (MS without Fe[II]-EDTA) for another 14 d, and the variations in leaf color were recorded. (**I**) Total chlorophyll accumulation (visualization of 0.4 g of plant leaves soaked in extraction solution and (**J**) content in leaves of WT, OE3, and OE5 under Fe-deficient conditions. (**K**) Visualization and (**L**) determination of root FCR activity in WT, OE3, and OE5 compared to that in WT under Fe-sufficient conditions. (**M**) The localization of root acidification activity and (**N**) root length analysis in WT, OE3, and OE5 under Fe-deficient conditions. Values shown are the mean ± SE (*n* = 3) of three biological replicates. Student’s *t*-test: Different lowercase letters above the columns indicate significant differences at *p* < 0.05. Bars = 1 cm.

We introduced *PbrSAUR72* into *S. lycopersicum* and analyzed the responses to Fe deficiency between them and the wild-type (WT). For transgenic plants to investigate the role of *PbrSAUR72* in FDR, *PbrSAUR72*-OE3 (OE3) and *PbrSAUR72*-OE5 (OE5) attracted our attention because of their high transcription levels (Figure S4A,B); therefore, they were selected for further study. As expected, all plants showed normal growth. We found no marked differences between the seedlings of WT, OE3, or OE5 under an Fe-sufficient condition, regardless of leaf color (Figure 4A,B,E) and root growth (Figure 4G). However, when exposed to a Fe-deficient medium, seedlings with *PbrSAUR72*-overexpression showed remarkably alleviation of the inhibition of root growth and true chlorotic leaves in WT plants under Fe-limited conditions (Figure 4H,N). They largely inhibited chlorophyll degradation (Figure 4I,L), implying that *PbrSAUR72* overexpression considerably maintained plant growth. Regarding the role of rhizosphere acidification and Fe reduction in Fe uptake, we subsequently investigated the capacities of WT, OE3, and OE5 under such conditions via root ferric chelate reductase (FCR) and pH colorimetric assays. Regarding the FCR activity in roots, no prominent differences were observed under Fe-sufficient conditions (Figure 4C,F). However, differences were observed in OE3 and OE5 compared to the WT under Fe-deficient conditions, as indicated by the deeper visible red in the FCR assay solution (Figure 4J). OE3 and OE5 displayed notably higher FCR activities (Figure 4M). Furthermore, no evident staining was observed in WT, OE3, or OE5 under Fe-sufficient conditions (Figure 4D). However, under Fe-deficient conditions, bright yellow staining was observed around the roots of *PbrSAUR72* overexpression lines. In contrast, light yellow staining was visible around those of the WT in the medium containing the pH indicator bromocresol purple (Figure 4K). This suggests that *PbrSAUR72* overexpression promotes rhizosphere acidification and Fe reduction, providing a favorable environment for Fe uptake.

**Figure 5:**
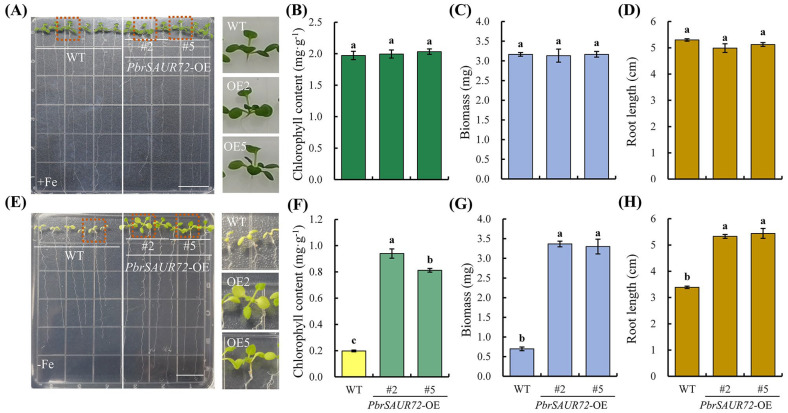
*PbrSAUR72* overexpression enhances the tolerance to Fe deficiency in *Arabidopsis* under Fe-deficient conditions. (**A**) Phenotypes of transgenic and wild-type (WT) *Arabidopsis* plants under Fe-sufficient conditions. The variations in the seedling leaf color of WT and transgenic line 2 (OE2) and 5 (OE5), cultured on MS medium for approximately three weeks, were recorded. (**B**) Total chlorophyll content, (**C**) biomass, and (**D**) root length of WT, OE2, and OE5 under Fe-sufficient conditions, respectively. (**E**) Phenotypes of transgenic and WT *Arabidopsis* plants under Fe-deficient conditions. Seedlings of WT and transgenic lines 2 (OE2) and 5 (OE5), cultured on MS medium for about 7 d, were transferred to a –Fe medium (MS without Fe[II]-EDTA) for another 14 d, and the variations in leaf color were recorded. (**F**) Total chlorophyll content, (**G**) biomass, and (**H**) root length of WT, OE2, and OE5 under Fe-deficient conditions, respectively. Values shown are the mean ± SE (*n* = 3) of three biological replicates. Student’s *t*-test: Different lowercase letters above the columns indicate significant differences at *p* < 0.05. Bars = 1 cm.

To further test the *PbrSAUR72* gene’s biological functions in FDR, we overexpressed *PbrSAUR72* in *A. thaliana*. We selected the transgenic lines *PbrSAUR72*-OE2 (OE2) and *PbrSAUR72*-OE5 (OE5) for Fe deficiency experiments based on their high expression levels of *PbrSAUR72* (Figure S4C,D). There were no marked differences between the WT and transgenic plants of *Arabidopsis* under normal conditions (Figure 5A–D). Nevertheless, under Fe-deficient stress, WT plants were more sensitive, developing Fe deficiency symptoms (Figure 5E). Furthermore, their chlorophyll content was significantly reduced (Figure 5F). In contrast, OE2 and OE5 exhibited green leaves with significantly higher chlorophyll content and biomass than WT plants (Figure 5E–G). In addition, WT plants showed significantly inhibited root growth in an Fe-deprived environment compared to both OE3 and OE5 plants (Figure 5H), illustrating the differences in the related biological processes within the root system. Generally, these results were consistent with the findings of the study on *S. lycopersicum*. These phenotypic and physiological differences highlighted *PbrSAUR72* as a positive regulator in improving plants’ tolerance to Fe deficiency.

### 2.5. Expression of Genes Related to Fe Deficiency Adaptive Response Were Elevated in PbrSAUR72-Overexpressing Transgenic Plants under Fe Deficiency Stress

**Figure 6:**
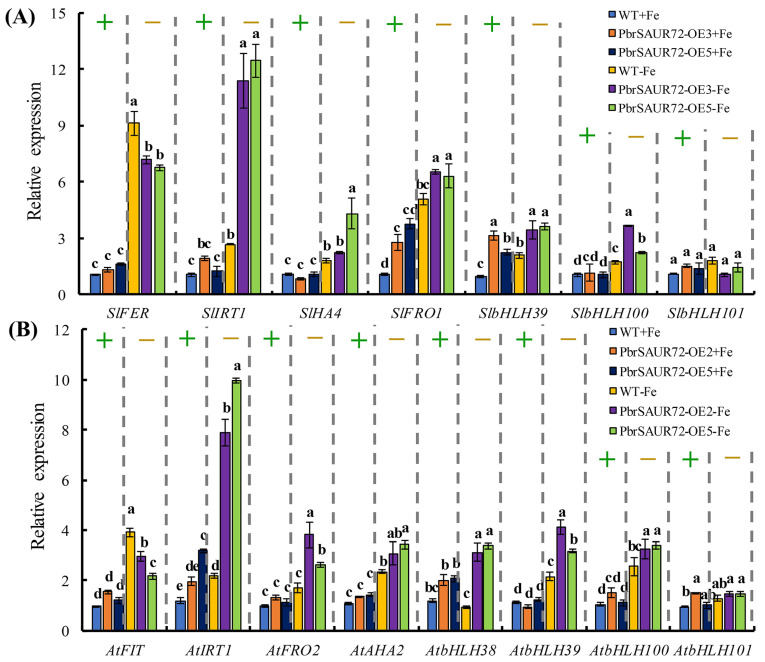
Expression profile of key genes involved in Fe deficiency responses in transgenic and wild-type (WT) tomato and *Arabidopsis* plants. (**A**) Seedlings of WT and transgenic tomato lines cultured on MS medium were shifted to a −Fe medium (MS without Fe[II]-EDTA) for another 14 d. The relative expressions of seven selected *PbrSAUR72*-regulated genes, *SlIRT1*, *SlFER*, *SlHA4*, *SlbHLH39*/*100*/*101*, and *SlFRO1* in the *PbrSAUR72*-overexpressing lines 3 (OE3) and 5 (OE5), compared to those in WT of *Solanum lycopersicum* under Fe-sufficient (+) and Fe-deficient conditions (−), were analyzed. (**B**) Seedlings of WT and transgenic *Arabidopsis* lines cultured on MS medium for 7 d were shifted to a –Fe medium (MS without Fe[II]-EDTA) for another 14 d. The relative expressions of eight selected *PbrSAUR72*-regulated genes, *AtIRT1*, *AtFER*, *AtHA4*, *AtbHLH38/39*/*100*/*101*, and *AtFRO1*, in the *PbrSAUR72*-overexpressing lines 2 (OE2) and 5 (OE5), compared to those in WT of *Solanum lycopersicum* under Fe-sufficient (+) and Fe-deficient conditions (−), were analyzed. The expression level detected in WT under Fe-sufficient conditions was used as the reference and set to ‘1’. *SlAcitn7* and *AtActin2* were used as internal controls. Values shown are the mean ± SE (*n* = 3) of three biological replicates. Student’s *t*-test: Different lowercase letters above the columns indicate significant differences at *p* < 0.05.

Strong FDR protects plants from Fe deficiency stress, and is governed by the expression of structural genes and some transcription factors, particularly bHLHs. Considering the enhanced acidification ability and strong Fe deficiency tolerance of *PbrSAUR72* overexpression lines (OE3 and OE5) when exposed to Fe starvation conditions, we hypothesized that *PbrSAUR72* might regulate the expression of Fe metabolism genes. To explore the effects of *PbrSAUR72* on the transcript levels of Fe-related genes, we chose several key Fe uptake-related genes, including *FER*/*FIT*, *IRT1*, *FRO*, *HA*, and bHLH Ib subgroup genes–bHLH38/39/100/101 (Figure 6A) for further study, and carried out qRT-PCR assays. In tomato, *SlFER*, *SlIRT1*, *SlHA4*, and *SlbHLH100* did not differ between WT, OE3, or OE5 under normal conditions. In contrast, *SlFRO1* and *SlbHLH39* were prominently expressed in *PbrSAUR72*-overexpressing lines. When challenged with Fe deficiency, all genes were activated, among which *SlIRT1* was expressed at far higher levels in OE3 and OE5. In addition, *SlHA4*, *SlFRO1*, *SlbHLH39*, and *SlbHLH100* were also highly expressed in OE3 and OE5 compared to WT. However, *SlHA4* was equivalently expressed *by* OE3 and WT. Furthermore, the identical subgroup genes of *SlbHLH39*, *SlbHLH100*, and *SlbHLH101* were not greatly stimulated by Fe deficiency stress, and changed negligibly between the *PbrSAUR72*-overexpressing transgenic and WT plants (Figure 6A). In short, *PbrSAUR72* overexpression integrally induced the expression of genes related to Fe reduction and uptake in tomatoes. The above results confirm the enhanced FCR and root acidification activities in *PbrSAUR72*-overexpressing tomato plants.

The results for *A. thaliana* were consistent with those obtained for tomatoes. No significant differences were observed in *AtFIT*, *AtFOR2*, *AtAHA2*, or *AtbHLH38*/*39*/*100* expression between transgenic (OE2 and OE5) and WT *Arabidopsis* plants under Fe-sufficient conditions (Figure 6B). Fe deficiency stimulated their expression, and the facilitation effect was more pronounced in OE2 and OE5, as indicated by their higher expression compared to the WT. In contrast, *AtFIT* presented the opposite trend. Likewise, *AtIRT1* was sensitive to Fe deficiency, and the upregulated expression of *AtIRT1* was detected in OE2 and OE5 under Fe-sufficient and Fe-deficient conditions compared to WT. Notably, *AtbHLH101* did not change obviously in OE2, OE5, or WT. It did not respond to Fe deficiency (Figure 6B), which was similar to the expression pattern observed for *SlbHLH101* in tomatoes under different levels of Fe content. Overall, the increased expression of Fe uptake-related genes may account for the enhanced Fe deficiency tolerance exhibited by both transgenic tomato and *Arabidopsis* plants under Fe-deprived conditions.

### 2.6. PbrSAUR72 Overexpression Enhanced Fe Accumulation under Fe-Limited Conditions

**Figure 7:**
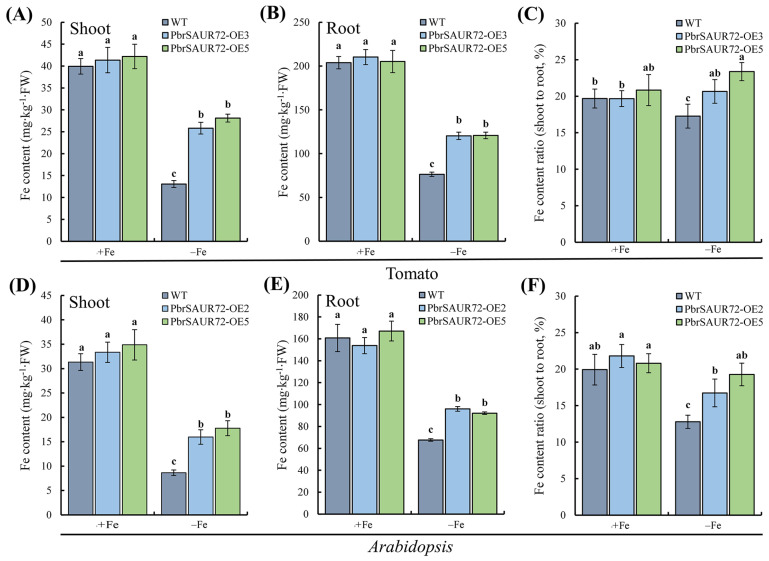
*PbrSAUR72* overexpression increases Fe accumulation and facilitates Fe transport from roots to shoots in both tomato and *Arabidopsis* under Fe-deficient conditions. Determination of Fe concentration in *PbrSAUR72*-overexpressing transgenic and wild-type (WT) plants of tomato and *Arabidopsis* under Fe-sufficient (+Fe) and Fe-deficient (−Fe) conditions. Seedlings of WT and transgenic lines cultured on MS medium for seven to ten days were shifted to −Fe medium (MS without Fe[II]-EDTA) for another 14 d, and the Fe accumulation in the roots and shoots of tomato (**A**,**B**) and *Arabidopsis* (**D**,**E**) were analyzed via inductively coupled plasma atomic emission spectrometry, respectively. In addition, the Fe content ratio of shoots to roots was calculated to assay the influences of *PbrSAUR72* on Fe transport in transgenic tomato (**C**) and *Arabidopsis* (**F**). The values shown are the mean ± SE (*n* = 3) of three biological replicates. Student’s *t*-test: Different lowercase letters above the columns indicate significant differences at *p* < 0.05.

Fe absorption by plants is mediated by FER/FIT, *FRO*, *IRT1*, etc. Our findings demonstrated that *PbrSAUR72*-overexpressing transgenic plants possessed greater Fe acquisition ability, as indicated by the stay-green phenotypes (Figure 4H and Figure 5E) and higher expression of Fe uptake-related genes (Figure 5A,B) under Fe deficiency. Subsequently, we investigated the Fe concentration in the roots and shoots of transgenic and WT tomato and *Arabidopsis* plants. We found no significant differences between transgenic and WT plants in the Fe content in either the roots or shoots under Fe-supplied conditions (Figure 7A,B,D,E). Conversely, the Fe concentration was significantly higher in the roots and shoots of transgenic plants than in WT under Fe-deficient conditions (Figure 7A,B,D,E). Moreover, we noticed that the Fe content ratio of shoots to roots was higher in transgenic plants, with no statistical difference under Fe-sufficient conditions. However, it displayed marked differences under Fe-deficient conditions compared to WT plants (Figure 7C,F). This suggests that *PbrSAUR72* overexpression promoted Fe uptake and transport from the roots to shoots under Fe-deficient conditions.

### 2.7. ROS and Hydrogen Peroxide (H_2_O_2_) Levels Were Decreased in PbrSAUR72-Overexpressing Plants under Fe-Deficient Stress

**Figure 8:**
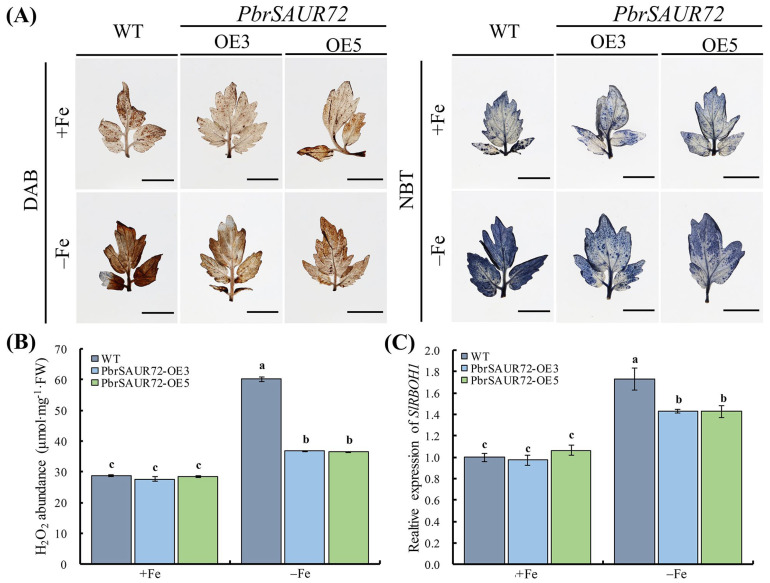
*PbrSAUR72* overexpression inhibited reactive oxygen species (ROS) accumulation in tomato under Fe-deficient conditions. (**A**) ROS accumulation analysis in *PbrSAUR72*-overexpressing and wild-type (WT) tomato plants under Fe-sufficient (+Fe) and Fe-deficient (−Fe) conditions. Seedlings of WT and transgenic lines 3 (OE3) and 5 (OE5) with primary roots approximately 2–3 cm long, cultured on MS medium, were shifted to −Fe medium (MS without Fe[II]-EDTA) for another 14 d. The ROS accumulation in the leaves was analyzed via 3,3-diaminobenzidine and nitro blue tetrazolium staining, respectively. Bars = 1 cm. (**B**) ROS abundance, in terms of H_2_O_2_, was detected in OE lines and WT under Fe-sufficient and Fe-deficient conditions by UV spectrophotometry. (**C**) The relative expression of a respiratory burst oxidase homologous gene, *SlRBOH1*, serving as a key regulator in the formation of ROS in plants, was detected. The expression level detected in WT under Fe-sufficient conditions was used as the reference and set to ‘1’. *SlActin7* was used as the internal control. Values shown are the mean ± SE (*n* = 3) of three biological replicates. Student’s *t*-test: Different lowercase letters above the columns indicate significant differences at *p* < 0.05.

ROS are known for their signaling function in controlling numerous biological processes and abiotic stresses, including Fe deficiency [32,33]. To test the hypothesis that enhanced Fe deficiency tolerance in *PbrSAUR72*-overexpressing tomato lines may be associated with ROS, we applied 3,3-diaminobenzidine (DAB) and nitro blue tetrazolium (NBT) as chromogenic substrates to detect the production of H_2_O_2_ and superoxide (O_2_^−^) anions, two of the principal ROS in WT and *PbrSAUR72*-overexpressing tomato lines (OE), under normal and Fe-deficient conditions. Under normal conditions, there was no evident difference in staining intensity between the OE lines and WT for either NBT or DAB. However, under Fe-limited conditions, less intense staining of DAB and NBT was observed in OE lines than in WT (Figure 8A), indicating a lower level of ROS production in *PbrSAUR72*-overexpressing tomato plants. Indeed, Fe deficiency-induced ROS abundance in H_2_O_2_, and, consistently, H_2_O_2_ content, exhibited no marked differences between OE lines and WT under Fe-sufficient conditions. However, they were all dramatically decreased in *PbrSAUR72*-overexpressing lines compared with WT when encountering Fe-limited stress (Figure 8B). Moreover, the expression of *SlRBOH1*, a key respiratory burst oxidase homologous gene that regulates the formation of ROS in tomato, prominently declined under Fe deficiency conditions compared to WT. At the same time, there was no significant variation between them when supplied with Fe (Figure 8C). Subsequently, the enhanced Fe deficiency tolerance was partially related to the inhibition of ROS production.

Finally, given the property that *PbrSAUR72* prefers to express in mature fruits (Figure 3D), we analyzed its function during fruit development and ripening. The results showed that *PbrSAUR72* overexpression did not significantly affect tomato plant growth or the fruit ripening process (Figure S5A–C). However, fruits of the transgenic lines at the mature green (MB) and mature red (MR) stages were significantly larger than the WT (Figure S5A,B), with a remarkably heavier single-fruit weight (Figure S5D) and vastly larger vertical and transverse diameters (Figure S5E,F). These results indicate a positive effect of *PbrSAUR72* on fruit development.

## 3. Discussion

Iron deficiency can contribute to chlorosis of young leaves and severely affect plant growth and development. Plants have evolved a complex regulatory network to maintain cellular Fe homeostasis in which molecular regulation and hormonal modulation are crucial [4,34]. Among all the phytohormones, auxin is thought to serve as a systemic signaler to trigger FDR. IAA could enhance root Fe(III) reductase activity, proton extrusion, and Fe uptake upon Fe deficiency via activating *FRO*, *IRT*, and *HA* expression [21]. IAA treatment can enhance the corresponding responses, while blocking IAA transport would severely affect FDR [22]. It can also integrate other major positive regulators of Fe deficiency, such as NO and ethylene, to jointly regulate FDR in *Arabidopsis* [35,36]. In addition, GABA-induced enhanced Fe deficiency tolerance in cucumber is also associated with increased IAA synthesis, signaling, and transport [23]. Thus, IAA is a positive factor in nutritional disorders, and a relatively high IAA content in leaves is conducive to the normal growth and development of plants exposed to Fe deficiency to a certain extent.

Our study observed evidence of similar roles of IAA in pear Fe deficiency. We noted that relatively lower Fe^2+^ content in leaves (Figure 1C) triggered leaf chlorosis (Figure 1A) by accelerating the chlorophyll degradation (Figure 1B) and IAA decomposition (Figure 1D), thus formatting CL. However, increased Fe^2+^ content in leaves (Figure 1C) triggered leaf regreening (Figure 1A) by accelerating chlorophyll and IAA synthesis (Figure 1B,D) in CL, thus formatting RL. In addition, although we posited that supplying IAA only at a concentration of 10 μM functioned and resulted in a light green appearance around the veins of CL (Figure 2E and Figure S1), it, indeed, dramatically augmented the transcription level of the chlorophyll synthesis genes in the CL (Figure 2F) and promoted the accumulation of Fe^2+^ (Figure 2G). This implied that a relatively higher IAA concentration in pear leaves might enhance FDR. Moreover, we detected enhanced IAA synthesis progress and a higher IAA content in the regreening spot with *PbrSAUR72* overexpression (Figure 2E–G), suggesting that increased IAA could enhance FDR by inducing chlorophyll synthesis and Fe transport and reduction. These findings confirm the positive regulatory role of IAA in FDR and further emphasize the critical importance of IAA in the FDR of pear plants.

Plants make a series of alterations in gene expression patterns to strengthen Fe acquisition and ensure their growth to a certain extent [13]. As one of the crucial downstream responsive families in auxin signaling, *SAURs* are required for auxin function. They are essential for hypocotyl elongation and development [37], as well as for adaption to plant growth under unfavorable environments, similarly to the positive roles of *AtSAUR4*, *AtSAUR32*, and *TaSAUR78* in the resistance to salt, drought, and freezing stress, respectively [30,38,39]. Therefore, *SAURs* play an important role in coping with abiotic stress. Here, we found that *PbrSAUR72* with RL expression preferences showed dominant differences from CL (Figure 1J,K; Table S2). We discovered a higher *PbrSAUR72* expression in roots that had undergone Fe deficiency, as well as for the other three genes (Figure 1J,K, Figure 2I and Figure S2A–F; Table S2). These results revealed that the SAUR genes studied herein are Fe deficiency-inducible, and their overexpression benefits FDR. Thus, we conclude that they are positive regulators of FDR in pear. In fact, transient overexpression of these four *PbrSAURs* within the CL of pears promotes chlorophyll synthesis at the injection area, resulting in the regreening phenotypes (Figure 2A–D), which is in line with our claim. Furthermore, given that this phenomenon was more pronounced with *PbrSAUR72* overexpression (Figure 2A) due to the gene’s function, but not the relative expression (Figure 2A–H and Figure S2G), we selected *PbrSAUR72* for further study.

We found that transiently overexpressing *PbrSAUR72* within the CL of pear might stimulate the progress of Fe transport and reduction, thus increasing the Fe^2+^ concentration (Figure 2H) in the regreening spot. In contrast, its transient silencing within the NL of pears might block this progress and resulting in chlorosis of NL (Figure S2A–F). This is consistent with the above findings. In addition, according to the results of the phylogenetic analysis, *PbrSAUR72* showed high homology to *AtSAUR72* and *AtSAUR41* (Figure 3B), which are reportedly active in the adaptation to adversity stress [30]. Thus, *PbrSAUR72* should be involved in adaptation to types of abiotic stress such as Fe deficiency.

It is widely known that Fe deficiency-tolerant plants are usually equipped with enhanced FDRs, such as promoting activity to acidify the soil and reducing ferric iron [40,41,42]. Soil acidification is essential and indispensable for improving plant resistance to Fe deficiency, and Fe^3+^ reduction is necessary for Fe utilization [13,14]; it could guarantee sufficient Fe^2+^ supply for root uptake [14]. In the subsequent study, we demonstrated that *PbrSAUR72* overexpression enhanced the tolerance of tomatoes and *Arabidopsis* to Fe deficiency. While it had no notable effects on their growth under normal conditions (Figure 4A–G and Figure 5A–D), the transgenic plants indeed grew better under Fe-limited conditions, as indicated by their greater biomass, decreased chlorophyll degradation, attenuated leaf chlorosis, and longer root growth. More importantly, the activity of FCR and the root acidification of transgenic lines were enhanced (Figure 4H–N and Figure 5E–H). These results are consistent with previous findings that it is the increased FCR activity and improved root acidification activity that account for the enhanced tolerance to *AtHAP5*/*MxMPK6-2*/*SlbHLH101* overexpression, positive regulators in FDR, lines to Fe deficiency [40,41,42]. Our study also revealed the positive role of *PbrSAUR72* in mediating Fe deficiency in pear plants.

Interestingly, we found that cytoplasm-localized PbrSAUR72, as key effector output of auxin signaling, negatively responded to IAA (Figure 3C). Its expression was significantly inhibited by IAA (Figure 2J), whereas its overexpression was instead able to promote the synthesis of IAA (Figure 2E). Hence, a plausible explanation for these results is that *PbrSAUR72*-regulated FDR may be independent of auxin signaling. Its overexpression can promote IAA synthesis. Apart from the above, we found that *PbrSAUR72* may be involved in fruit development. We observed a higher expression level of *PbrSAUR72* in mature pear fruit than in tender tissues with weaker expression (Figure 3D), and its ectopic overexpression was indeed able to promote the fruit development of tomatoes without effects on plant growth or fruit ripening (Figure S5). However, auxin is known to be found at low levels in fruits in the middle and late development stages (including mature fruit) as a result of blocked synthesis [43] and accelerated degradation under the action of ET or other factors [44]. This further suggests its negative correlation with IAA signaling, and corresponds to its function as an IAA signaling repressor. Collectively, we defined *PbrSAUR72* as an IAA-negative responsive factor, and it was at least partly able to facilitate fruit development and strengthen Fe deficiency tolerance in an IAA signaling-independent manner.

Fe deficiency responses are controlled by an intricate regulatory network. The bHLH transcription factor, *LeFER*, a core signaling regulator identified in tomato, as well as its Arabidopsis ortholog, *AtFIT*, are induced by Fe deficiency and are crucial for activating the expression of downstream genes to ensure Fe absorption under Fe-limited conditions [45,46,47]. For example, *LeFER* (*SlFER*) or *AtFIT* plants experiencing loss of function have no ability to stimulate the expression of genes responsible for Fe reduction and root acidification, displaying extreme Fe-deficient phenotypes due to the dominantly decreased Fe content in both roots and shoots [45,46]. However, in *Arabidopsis*, singular *AtFIT* overexpression makes it difficult to maintain constitutive and strong responses to Fe deficiency [46,48]. *AtFIT* is said to be a transcriptional activator that lacks DNA-binding activity [49]. Members of the bHLH Ib subgroup, triggered by Fe deficiency, are the main drivers for its binding to the promoters of *AtIRT1* and *AtFRO2* by interacting with FIT, including bHLH38/39/100/101 [49,50,51]. In addition, the higher expression of Fe uptake and transport genes is not sufficient to cope with Fe-shortage. Soil acidification is a prerequisite step for improving plant resistance to Fe deficiency [52], which, in the *AtAHA2* regulation, is a plasma membrane gene invoked by Fe deficiency. This emphasizes that increased gene expression in the Fe acquisition system is responsible for Fe absorption and resistance to Fe deficiency. In contrast, the induction of bHLH Ib and *AHA2* is necessary to manage Fe deficiency. In this study, we discovered that Fe deficiency induced *SlFER*, *SlIRT1*, *SlFRO1*, *SlHA4*, *SlbHLH39*, and *SlbHLH100* expression in tomatoes and stimulated *AtFIT*, *AtIRT1*, *AtAHA2*, *AtbHLH39*/*100* in *Arabidopsis*. In addition, a considerable increase in *SlIRT1*, *SlFRO1*, *SlHA4*, *SlbHLH39*, and *SlbHLH100* in *PbrSAUR72*-overexpressing transgenic tomato plants (Figure 6A) and a significant upregulation of *AtIRT1*, *AtAHA2*, and *SlbHLH38*/*39*/*100* in *PbrSAUR72*-overexpressing transgenic *Arabidopsis* plants were also determined compared to WT in a Fe-deficient environment (Figure 6B). These results demonstrate the positive role of *IRT1*, *HA*, *FRO*, and *bHLH39*/*100* in FDR. The results can explain the enhanced FCR activity (Figure 4J,M), root acidification (Figure 4K), and higher Fe content (Figure 7) in transgenic tomato plants under Fe-deficient stress, which are fully consistent with previous studies [53,54,55]. Notably, we observed prominently higher *SlFER* and *AtFIT* expression in the WT of both tomato and *Arabidopsis* (Figure 6A,B); however, the FDR was, indeed, somewhat weaker than that in transgenic plants (Figure 4H–N, Figure 5E–H and Figure 6A,B). Based on previous r results [46,48,49,50,51], a reasonable explanation for this phenomenon is that the lower *bHLH39* and *bHLH100* expression within WT limits the DNA-binding activity of *SlFER* and *AtFIT* to the promoter of *IRT1* and *FRO1*, thus impairing the expression intensity of Fe acquisition and reduction genes and reducing tolerance to iron deficiency, which can be reflected by the *IRT* and *FRO* expression in plants (Figure 6A,B). In summary, the enhanced tolerance of *PbrSAUR72*-overexpressing lines to Fe deficiency is the result of the markedly higher expression of Fe-induced genes when subjected to Fe deprivation stress. Based on the higher Fe content ratio of the shoots to the roots of transgenic plants (Figure 7C,F), we can deduce that *PbrSAUR72* facilitates the transport of Fe from the roots to the shoots.

Reactive oxygen species are considered as vital secondary signaling agents in plants, and are involved in regulating several physiological processes [32,33]. ROS are the most common toxic byproducts of abiotic stress [56,57], and are strictly controlled by the NADPH oxidase-mediated pathway, encoded by the respiratory burst oxidase homologous genes (*RBOH*) [58]. Fe deficiency induces ROS production [59], and excessive ROS disrupts the electron transport chain and triggers oxidative damage, and even programmed cell death, to plants [57]. Therefore, ROS homeostasis is crucial. In this study, we observed intense light staining in transgenic and WT tomatoes under normal conditions via DAB and NBT staining, but darker staining under Fe-deficient conditions. Notably, less intense staining was found in transgenic plants than in WT plants when subjected to Fe deficiency (Figure 8A). Fe deficiency induced H_2_O_2_ accumulation and upregulated *SlRBOH1* expression, although both were at a lower level in transgenic plants than in WT under the same Fe-deficient conditions (Figure 8B,C). These results imply that Fe deficiency triggers ROS accumulation in plants and *PbrSAUR72* overexpression eliminates excessive ROS induced by Fe deficiency, relieving plants from oxidative stress caused by excessive ROS production.

Collectively, *PbrSAUR72*-regulated tolerance to Fe deficiency stress is associated, at least partly, with ROS signaling and the regulation of Fe metabolism-related genes via IAA-independent pathways. Under this dual action, the Fe concentration in the roots and leaves, as well as the transport of Fe content from the roots to shoots of the *PbrSAUR72*-overexpressing transgenic plants under Fe-deficient stress, increased significantly (Figure 7), ensuring their development (Figure 4H–N and Figure 5E–H).

## 4. Materials and Methods

### 4.1. Plant Materials, Growth Conditions, and Treatments

The experiment was conducted in May 2020. The chlorotic leaves (CLs) used in this work were collected from 55-year-old ‘Dangshansuli’ pear (*P. bretschneideri* Rehd.) trees grafted onto ‘Duli’ pear (*P. betulaefolia* Bunge.) rootstock in the Old Yellow River Valley region of Dangshan County, Anhui Province (China). In this area, the soil is primarily alkaline due to improper management and abuse of fertilizers; consequently, chlorosis of pear trees easily occurs. The RL was obtained by spraying 0.2% FeSO_4_ solution on CL, and the RL used for analysis consisted of the leaves collected at 9 d after treatment. The trees used here were pollinated artificially with ‘Yali’ pear (*P. bretschneideri*) pollen at the first open bloom. All trees were positioned and pruned in an open area and spaced 4 m apart within rows at 6 m intervals, with crops in between them. The Fe^2+^ and IAA content of RL and CL were detected prior to the subsequent experiments. The transient transformation of *PbrSAURs* overexpression was conducted on CL, and the transient silencing of *PbrSAUR72* was carried out on the normal leaves (NLs) of pear plants using sterile injectors. After 14 days of infection, photographs were taken with a digital camera (EOS 80D, Cannon, Beijing, China). The chlorophyll content or SPAD values of the injected spots were determined in order to reflect the leaf color variation. Before this step, the leaf spots after 7 d of treatment were collected and stored at −80 °C for expression analysis of the genes involved in chlorophyll synthesis (*PbrPOR*, *PbrPOR-like*, *PbrCHL*, *PbrCHL-like*, and *PbrCAO*), chlorophyll degradation (*PbrPAO*, *PbrCLH*, *PbRCCR*), and key enzyme activities related to IAA synthesis (indolealdehyde oxidase [AAldO] and 3-indolepyruvate decarboxylase [IPDC]), respectively. In addition, the soil along the main roots of the pear trees was removed with a stainless steel shovel until the absorption roots were exposed in order to explore the expression of related genes in the roots of both normal and chlorotic pear trees. The surface soil on the absorption roots was then cleaned with a brush and washed 2–3 times with distilled water. Finally, 10 g samples of the absorption roots were placed onto sterile filter paper to remove surface water, immediately frozen in liquid nitrogen, and stored at −80 °C until analysis.

Wild-type *A. thaliana* and tomato (*S. lycopersicum*) *Micro*-*Tom* and transgenic lines overexpressing *PbrSAUR72* in the corresponding background were cultivated in an environmentally controlled growth room under long-day conditions, with a 16 h light (25 °C ± 1 °C)/8 h dark (25 °C ± 1 °C) cycle and 80% relative humidity, as well as a 12,000 lux light intensity. *Arabidopsis* seeds were surface-sterilized in 75% alcohol with 0.1% (*v*/*v*) Tween 20 for 5 min and soaked in 95% alcohol for 1 min. They were washed 3–4 times with distilled water, and the liquid was discarded. Finally, they were placed on sterile filter paper for approximately 2 min to remove residual water, then germinated on MS Base Salts (Coolaber Technology Co., Ltd., Beijing, China. PM1011) supplemented with 3% sucrose (*w*/*v*) and cytoplasm-localized 1.0% (*w*/*v*) agar adjusted to pH 5.8. Seeds were vernalized for two days in a refrigerator (4 °C) in darkness and then placed into a culture room at 25 °C. For tomato seed sterilization, 20% sodium hypochlorite solution was used for approximately 15 min, and vernalization at 4 °C was replaced by darkness treatment. Other operations were applied as for *A. thaliana* above.

For FDR analysis, the experiments were carried out as previously described [40,60], with some modifications. Briefly, surface-sterilized tomato seeds were sown on an MS medium and germinated. Then, seedlings with approximately 2–3 cm long primary roots grown on MS medium (for approximately 10 days) were transferred to a medium with Fe (Fe-sufficient (+Fe), MS media as a control) or without Fe (Fe-deficient (–Fe), MS without Fe(II)-EDTA) and grown under the above growth conditions for another 14 days. For *Arabidopsis*, surface-sterilized *Arabidopsis* seeds were grown on MS medium and germinated for 7 days on MS. Uniform seedlings were shifted to +Fe medium or –Fe medium for another 14 days. Similarly, the phenotypes were recorded using a camera, and relevant metrics were measured. All experiments were performed independently in triplicate, and more than nine independent lines were used for each measurement.

For the fruit development assay, two-week-old transgenic and WT tomato seedlings grown on MS medium were transplanted into pots containing culture substrates (1:1 nutrient soil/vermiculite) and placed in an environmentally controlled growth room under long-day conditions at 25 °C. Plant growth and fruit development were observed following this. The plant height and fruit characteristics of transgenic and WT tomatoes at the mature green stage were compared to explore the effect of *PbrSAUR72* on plant growth, fruit development, and fruit ripening. The signal fruit weight and the vertical and transverse diameters of fruits at the mature red stage were detected using an electronic balance (PX224ZH, OHAUS Co., Ltd., Shanghai, China) and a vernier caliper (111-101-10G, Guanglu Measuring Instrument Co., Ltd., Guilin, China).

Exogenous 0.2% FeSO_4_ treatment was conducted on CL at nightfall on a sunny day as previously described [31]. Briefly, 20 g samples of the substances were fully dissolved with 10 L of distilled water via a backpack sprayer. The solution was sprayed evenly on CL until the mist droplets on the leaves condensed into water droplets and dropped naturally. Representative images were taken with a camera at 0, 3, 6 9, and 12 d after treatment. CL treated with distilled water was used as the control. The leaf samples of CL and RL after 6 d of treatment were collected for analysis of the chlorophyll, IAA, and Fe^2+^ levels. For exogenous IAA treatment, 2.5, 5, 10, and 20 μM IAA (YUANYE Biotechnology Co. Ltd., Shanghai, China) was sprayed evenly onto the CL using a spray pot. IAA was dissolved in water-free alcohol and then diluted with distilled water to the appropriate concentration. The experiment was performed at nightfall on a sunny day. In the following days, changes in leaf color were observed and recorded using a camera when the CL showed significant changes (approximately 14 d after treatment), and the chlorophyll content was determined. The CL treated with distilled water was used as the control. Subsequently, the samples at 7 d after treatment were analyzed for Fe^2+^ content and expression of chlorophyll synthesis-related genes. To explore the response of *PbrSAUR72* to IAA, 10 μM IAA was sprayed evenly onto NL using a spray pot. Leaves from 0, 0.5, 1, 2, and 4 h after IAA treatment within three biological replicates were cut using scissors, immediately frozen in liquid nitrogen, and stored at −80 °C for gene expression analysis.

### 4.2. Determination of IAA Content, Enzyme Activities, and Fe Concentration

The IAA contents in RL, CL, and the respective spots after injection with empty vector and *PbrSAUR72* overexpression were measured via enzyme-linked immunosorbent assay (ELISA) [61]. Briefly, samples were thawed and kept at 4 °C. A certain amount of PBS solution (at pH 7.4) was added and ground into homogenates using a cooled glass homogenizer, followed by centrifugation at 3000 rpm for 20 min. The supernatant was collected and analyzed according to the manufacturer’s instructions (ml147100-2; Shanghai Enzyme-linked Biotechnology Co., Ltd., Shanghai, China). Correspondingly, the activities of two enzymes involved in the IAA generation of injected spots, IAAldO and IPDC, were detected based on the methodology of Bower et al. [62] and Koga et al. [63], respectively.

For Fe detection in RL and CL, samples were cleaned with 0.1% (*w*/*v*) sodium dodecyl sulfate and washed with distilled water three times. Samples (1.0 g) were wiped dry, cut into pieces with plastic scissors, and extracted with 0.1 M HCl (50 mL) for 24 h in the dark. Next, extracts (5 mL) were transferred to 45 mL of assay solution containing 0.4% (*w*/*v*) hydroxylamine hydrochloride, 1% (*w*/*v*) sodium acetate, and 0.004% (*w/v*) 1,10–phenanthroline (with 0-, 1-, 2-, 3-, 4-, 5- mg∙L^−1^ ammonium ferrous sulfate dodecahydrate as the standard). Finally, the reaction mixture was placed in the dark for 30 min, and the Fe(II)-ferrozine complex absorbance of each assay solution was determined using a spectrophotometer (V-T3/V-T3C; Yipu Instrument Manufacturing (Shanghai) Co., Ltd., Shanghai, China) at 560 nm [64]. In addition, inductively coupled plasma atomic emission spectrometry (ICP-AES, AIRIS/AP, TJA, Thermo Fisher Scientific, Shanghai, China) was used to assess the Fe concentration in seedlings. Tomato and *Arabidopsis* seedlings were separated into roots and shoots with three biological replicates after +Fe and –Fe treatments. The samples were transferred to a 60 °C environment for 3 d to remove moisture, and the dried tissues were ground into powder (KZ-III; Wuhan Servicebio Technology Co., Ltd., Wuhan, China). Subsequently, the samples were weighted, and 0.1 g powder was added into 5 mL HNO_3_ for 30 min. The samples were digested at 180 °C for 25 min prior to Fe measurement [65].

### 4.3. FCR and Rhizosphere pH Location Assays

Root FCR activity was measured and visualized as previously described by Aksoy and Koiw [66], with some optimizations. In brief, freshly excised tomato roots from each sample were immersed in 1 mL FCR assay buffer solution (0.1 mM Fe(III)-EDTA and 0.3 mM Ferrozine) in a 1.5 mL Eppendorf tube (pH 5.5) for approximately 1 h in the dark. An assay solution without roots was used as the control, and the absorbance of each tube was determined with a spectrophotometer at 562 nm. The data are described as root FCR activity relative to WT under Fe-sufficient conditions (%). For in situ localization of root pH, freshly excised tomato roots from each sample were placed into an assay medium consisting of 1 mM CaSO_4_ and 0.006% (*w*/*v*) pH indicator (bromocresol purple) with 0.7% (*w*/*v*) agar for 1 h in the dark before color formation was photographed with a digital camera [67].

### 4.4. Chlorophyll Content, Root Length, and ROS Accumulation Assays

To analyze the chlorophyll content, 0.2 g samples of plant leaves were cut into pieces with plastic scissors and soaked in an extraction solution (50% (*v*/*v*) acetone + 40% (*v*/*v*) absolute alcohol) for 24 h. The absorbance of each extract was detected with a spectrophotometer at 663 nm and 645 nm, respectively. Chlorophyll content formulas were used as previously described, with some modifications [68]. A portable chlorophyll meter (SPAD-502 Plus, KONICA, Tokyo, Japan) was used to determine the SAPD value to reflect the total chlorophyll content of NL-silencing *PbrSAUR72*. Additionally, ROS accumulation in transgenic and WT tomato plants under Fe-sufficient and Fe-deficient conditions was visualized via DAB and NBT staining, as described by Kumar et al. [69]. Moreover, ROS in terms of H_2_O_2_ were qualitatively analyzed as described by Lin and Kao [70], with some modifications. Briefly, 1 g of each tomato leaf sample was ground in a precooled acetone solution (5 mL) in an ice bath, followed by centrifugation at 12,000 rpm for 20 min at 4 °C. The supernatant was collected, and the reaction solution was prepared according to the manufacturer’s instructions. Finally, the absorbance was determined at 415 nm, and the H_2_O_2_ content was estimated by using the standard curve. For the root length analysis, we used ImageJ version 1.8.0.

### 4.5. Phylogenetic Analysis and Sequence Alignment

The *PbrSAUR72* sequence was downloaded from the NCBI search database (https://www.ncbi.nlm.nih.gov/, accessed on 3 May 2021), and the sequence of *AtSAUR* genes was obtained from the *Arabidopsis* Information Resource (TAIR, https://www.arabidopsis.org/, accessed on 5 May 2021). Multiple sequence alignments were performed based on the auxin-inducible domain of the protein sequences with the optimal parameters, including P-distance and pairwise deletion. ClustalW 2.0 software was used to develop the multiple sequence alignments. MEGA software (version 7.0) was used to construct and visualize a maximum likelihood (ML) phylogenetic tree with a bootstrap value of 1000, in which the neighbor-joining test was the best substitution model. PowerPoint 2016 was used for the annotation and review of the phylogenic tree. Multiple alignments of the homologous amino acid sequences of *PbrSAUR72* from *M. domestica* and *A. thaliana* were performed and visualized using the DNAMAN software version 8.0.

### 4.6. Plasmid Construction and Pear Leaf Transient Transformation

Primers for *Pbr033623.1* (*PbrSAUR72*), *Pbr022379.1*, *Pbr013531.1*, and *Pbr029068.1* from cultivated pear (‘Dangshansuli’) were designed based on their sequences in the NCBI database (https://www.ncbi.nlm.nih.gov/, accessed on 3 May 2021). Taking *PbrSAUR72* as an example, the coding sequence of *PbrSAUR72* (without TAG) cloned from CL was constructed into the p1300U1-Flag vector digested with *BamH* I and *SaI* I sites, using primers *PbrSAUR72*-Flag-F and *PbrSAUR72*-Flag-R (Table S1) to generate the overexpression fusion vector, *PbrSAUR72*-Flag, under the control of the CaMV 35S promoter. For virus-induced silencing of *PbrSAUR72* in pear, tobacco rattle virus (TRV) was introduced, as it effectively infects pear plants [71]. Approximately 400 bp samples of the *PbrSAUR72* gene-specific regions were inserted into a pTRV2 vector digested with *BamH* I and *EcoR* I sites using the primers *PbrSAUR72*-TRV-F and *PbrSAUR72*-TRV-R (Table S1) to generate the silencing fusion vector, *PbrSAUR72*-TRV, along with the strong 35S promoter upstream. pTRV1 was used as an auxiliary vector. The recombinant vectors were transformed into the *Agrobacterium tumefaciens* strain GV3101 via thermal stimulation, which was then infiltrated into the leaves of ‘Dangshansuli’ pear in the field. The suspension used in this experiment contained 10 mM MgCl_2_, 4 mM 4-morpholineethanesulfonic acid, and 0.2 mM acetosyringone. After 14 days, changes in the leaf colors of the spots were recorded using a camera, which was used for further IAA analysis and Fe^2+^ analysis.

### 4.7. Plant Transformation and Subcellular Localization

To generate overexpressing plant lines, the fusion vector *PbrSAUR72*-Flag, which was transformed into *Ag. tumefaciens* strain GV3101, was used. Genetically modified *Arabidopsis* and tomato plants were obtained using the *Agrobacterium*-mediated leaf disk transformation method [72] and *Agrobacterium*-mediated floral dip [73]. Both transgenic lines were selected and confirmed at the DNA and transcriptional levels for further analysis. Two independent transgenic lines were selected, and all experiments were performed using homozygous lines of the T3 generation which had been screened for their resistance to hygromycin B.

For subcellular localization of PbrSAUR72, the full coding sequence cloned from the CL of *PbrSAUR72* (without TAG) was inserted into the *Xba* I and *BamH* I sites of the antisense strand N-terminus of the p1300-35S-GFP-BS2 vector to generate the *PbrSAUR72*-GFP fusion constructs. The empty vector (control) and *PbrSAUR72*-GFP fusion constructs were then transformed into the tobacco leaf epidermis of 30-day-old plants via *Agrobacterium*-mediated transformation. After *agro*-infiltration for 48 h, the protein expression of the empty vector or *PbrSAUR72*-GFP from the leaves was visualized using a confocal laser scanning microscope (FV1000; Olympus Corporation, Tokyo, Japan) with an emission wavelength of 395 nm.

### 4.8. RNA Extraction and qRT-PCR Analysis

Total RNA and the corresponding first-strand cDNA of each sample were extracted and synthesized with the RC411-01 and R312-01 (Vazyme Biotechnology Co., Ltd., Nanjing, China) kits. The samples were stored at −20 °C for further analysis. Subsequently, qRT-PCR was performed to detect *PbrSAURs* expression levels in the RL, CL, and other tissues of ‘Dangshansuli’ pear plants. Additionally, the expression of chlorophyll synthesis-related genes in regreening spots overexpressing *PbrSAUR72* was counted by applying qRT-PCR. Fe metabolism-associated gene expression levels of *PbrSAUR72*-overexpressing transgenic and WT plants under +Fe and –Fe conditions were assayed via qRT-PCR, and the transgenic plants were also identified using this method. We used Primer Premier software (version 6.0) to design gene-specific primers (Table S1). The qRT-PCR assay was performed on an ABI StepOne qRT-PCR cycler (Applied Biosystems, Foster City, CA, USA), with three biological replicates performed per treatment. The procedure was as follows: 10 μL SYBR Green Master Mix (Q131-02, Vazyme Biotechnology Co., Ltd., Nanjing, China), 6.4 μL DEPC, 0.8 μL upstream and downstream primers, and 2 μL cDNA (400 ng). The program for the thermocycler consisted of pre-denaturation at 95 °C for 5 min, 40 cycles of denaturation at 95 °C for 10 s, annealing at 60 °C for 20 s, extension at 72 °C for 20 s, and final extension at 72 °C for 2 min. The 2^−∆∆CT^ method was used to calculate the relative gene expression [74]. Pear *PbrGAPDH*, *Arabidopsis AtActin2*, and tomato *SlActin7* were used as internal controls (Table S1).

### 4.9. Statistical Analysis

All data are presented as mean ± standard error (SE). Pairwise comparisons were computed to determine whether they were significantly different from each other using Student’s *t*-test, as indicated by different lowercase letters. Data were analyzed using Microsoft Excel software (Version 2017), and SPSS Version 18.0 for Windows (SPSS Inc., Chicago, IL, USA) was used to analyze significant differences.

## 5. Conclusions

**Figure 9:**
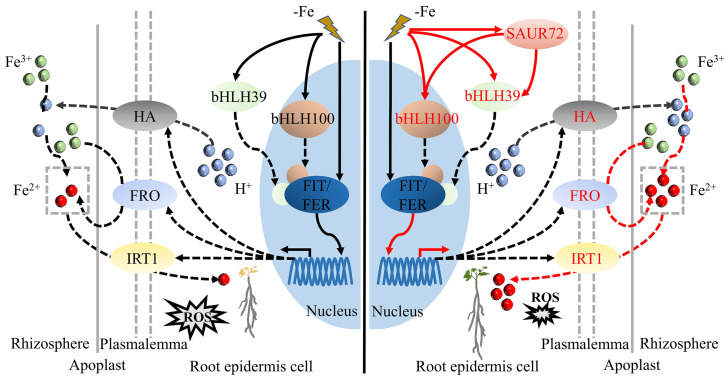
A working model for the role of the *PbrSAUR72* gene in regulating Fe deficiency tolerance. Fe deficiency induces the expression of bHLH39, bHLH100, and the related downstream genes, such as *IRT1*, *FRO*, and *HA*. More importantly, *PbrSAUR72* overexpression optimizes this promoting effect and enhances iron reduction and uptake. It inhibits reactive oxygen species (ROS) accumulation and facilitates Fe accumulation in both roots and leaves, maintaining normal plant growth under Fe-deficient conditions. Solid lines indicate transcriptional activation and dashed lines indicate transport or reduction processes. Red fonts and red lines indicate the increase in and enhancement of the related protein and process, respectively.

In conclusion, we proposed a model for the protective functions of *PbrSAUR72* against Fe deficiency stress in pears. As described, *PbrSAUR72* overexpression optimized the promoting effect of Fe acquisition-related genes by Fe deficiency, and was equipped with a complete Fe reduction and uptake system. Beyond this, it inhibited ROS production and ensured an adequate Fe supply to the plants, maintaining their normal growth under Fe-deficient environments (Figure 9). These findings could offer a theoretical basis for breeding Fe deficiency-tolerant projects and provide insights to enrich the regulatory network for Fe deficiency. However, as we were limited by the existing experimental conditions, we were unable to further study the function of *PbrSAUR72* in FDR using the corresponding mutants in *Arabidopsis* or tomato, which is a key breakthrough which we will attempt to make soon. Based on the results, we intend to construct a yeast library of pear cDNA and carry out yeast library screening, aiming to reveal the detailed mechanism underlying it.

## Data Availability

The data that support the results of this study are available from the corresponding author upon reasonable request.

## Supplementary Materials

The following supporting information can be downloaded at: https://www.mdpi.com/article/10.3390/plants12112173/s1, Figure S1: Representative images of CL treated with different doses of indoleacetic acid (IAA); Figure S2: *PbrSAUR* expression analysis in leaf and roots of pear trees; Figure S3: Transiently silencing *PbrSAUR72* aggravates chlorophyll degradation and receding Fe^2+^ accumulation in normal leaf of pear (*Pyrus bretschneideri* Rehd.); Figure S4: Molecular identification of *PbrSAUR72*-OE transgenic plants; Figure S5: Representative images of whole plants and fruits for transgenic and wild-type (WT) tomato plants; Table S1: The list of primers used in this study for qRT-PCR and vectors construction; Table S2: Transcriptome analysis of differentially expressed genes (DEGs) of *SAUR* in regreening leaf (RL) and chlorotic leaf (CL) of pear.

## References

[1] Guerinot M.-L., Yi Y. (1994). Iron: Nutritious, noxious, and not readily available. Plant Physiol..

[2] Briat J.-F., Dubos C., Gaymard F. (2015). Iron nutrition, biomass production, and plant product quality. Trends Plant Sci..

[3] Xiao Q., Zhong Y., Lu S. (2015). Assessment of heavy metal pollution and human health risk in urban soils of steel industrial city (Anshan), Liaoning, Northeast China. Ecotox. Environ. Safe.

[4] Curie C., Mari S. (2017). New routes for plant iron mining. New Phytol..

[5] Alvarez-Fernandez A., Melgar J.-C., Abadia J., Abadia A. (2011). Effects of moderate and severe iron deficiency chlorosis on fruit yield, appearance and composition in pear (*Pyrus communis* L.) and peach (*Prunus persica* (L.) Batsch). Environ. Exp. Bot..

[6] Brumbarova T., Bauer P., Ivanov R. (2015). Molecular mechanisms governing *Arabidopsis* iron uptake. Trends Plant Sci..

[7] Merry R., Dobbels A.-A., Sadol W., Naeve S., Stupar R.-M., Lorenz A.-J. (2022). Iron deficiency in soybean. Crop Sci..

[8] Li Q., Chen L., Yang A. (2020). The molecular mechanisms underlying iron deficiency responses in rice. Int. J. Mol. Sci..

[9] Valentinuzzi F., Venuti S., Pii Y., Marroni F., Cesco S., Hartmann F., Mimmo T., Morgante M., Pinton R., Tomasi N. (2019). Common and specific responses to iron and phosphorus deficiencies in roots of apple tree (*Malus* × *domestica*). Plant Mol. Biol..

[10] Zamboni A., Zanin L., Tomasi N., Pezzotti M., Pinton R., Varanini Z., Cesco S. (2012). Genome-wide microarray analysis of tomato roots showed defined responses to iron deficiency. BMC Genom..

[11] Briat J.-F., Fobis-Loisy I., Grignon N., Lobreaux S., Pascal N., Savino G., Thoiron S., Wiren N., Wuytswinkel O. (1995). Cellular and molecular aspects of iron metabolism in plants. Biol. Cell.

[12] James M.-C., Janneke B., Jorge R.-C. (2017). Iron homeostasis in plants—A brief overview. Metallomics.

[13] Ivanov R., Brumbarova T., Bauer P. (2012). Fitting into the harsh reality: Regulation of iron-deficiency responses in dicotyledonous plants. Mol. Plant.

[14] Robinson N.-J., Procter C.-M., Connolly E.-L., Guerinot M.-L. (1999). A ferric-chelate reductase for iron uptake from soils. Nature.

[15] Vert G., Grotz N., Dedaldechamp F., Gaymard F., Guerinot M.-L., Briat J.-F., Curie C. (2002). IRT1, an *Arabidopsis* transporter essential for iron uptake from the soil and for plant growth. Plant Cell.

[16] Tong H., Madison I., Long T.-A., Williams C.-M. (2020). Computational solutions for modeling and controlling plant response to abiotic stresses: A review with focus on iron deficiency. Curr. Opin. Plant Biol..

[17] Tissot N., Robe K., Gao F., Grant-Grant S., Boucherez J., Bellegarde F., Maghiaoui A., Marcelin R., Izquierdo E., Benhamed M. (2019). Transcriptional integration of the responses to iron availability in *Arabidopsis* by the bHLH factor ILR3. New Phytol..

[18] Gao F., Robe K., Gaymard F., Izquierdo E., Dubos C. (2019). The transcriptional control of iron homeostasis in plants: A tale of bHLH transcription factors. Front. Plant Sci..

[19] Riaz N., Guerinot M.-L. (2021). All together now: Regulation of the iron deficiency. J. Exp. Bot..

[20] Qian Y.-C., Zhang T.-Y., Yu Y., Gou L.-P., Yang J.-T., Xu J., Pi E.-X. (2021). Regulatory mechanisms of bHLH transcription factors in plant adaptive responses to various abiotic stresses. Front. Plant Sci..

[21] Wu T., Zhang H.-T., Wang Y., Jia W.-S., Xu X.F., Zhang X.-Z., Han Z.-H. (2012). Induction of root Fe(III) reductase activity and proton extrusion by iron deficiency is mediated by auxin-based systemic signaling in *Malus xiaojinensis*. J. Exp. Bot..

[22] Bacaicoa E., Mora V., Zamarreno A.-M., Fuentes M., Casanova E., Garcia-Mina J.-M. (2011). Auxin: A major player in the shoot-to-root regulation of root Fe-stress physiological responses to Fe deficiency in cucumber plants. Plant Physiol. Biochem..

[23] Guo Z.-X., Du N.-X., Li Y.-N., Zheng S.-X., Shen S.-S., Piao F.-Z. (2020). Gamma-aminobutyric acid enhances tolerance to iron deficiency by stimulating auxin signaling in cucumber (*Cucumis sativus* L.). Ecotox. Environ. Safe.

[24] Sun H.-W., Feng F., Liu J., Zhao Q.-Z. (2018). The interaction between auxin and nitric oxide regulates root growth in response to iron deficiency in rice. Front. Plant Sci..

[25] Hou K., Wu W., Gan S.-S. (2013). *SAUR36*, a small auxin up gene, is involved in the promotion of leaf senescence in *Arabidopsis*. Plant Physiol..

[26] Kant S., Bi Y.-M., Zhu T., Rothstein S.-J. (2009). *SAUR39*, a small auxin up RNA gene, acts as a negative regulator of auxin synthesis and transport in rice. Plant Physiol..

[27] Bemer M., Van M.-H., Muino J.-M., Ferrandiz C., Kaufmann K., Angenent G.-C. (2017). FRUITFULL controls *SAUR10* expression and regulates *Arabidopsis* growth and architecture. J. Exp. Bot..

[28] Wen W.-Z., Mei Y.-Y., Zhou J., Cui Y.-J., Wang D., Wang N.-N. (2020). *SAUR49* can positively regulate leaf senescence by suppressing *SSPP* in *Arabidopsis*. Plant Cell Physiol..

[29] Wang Z., Yang L., Liu Z., Lu M., Wang M., Sun Q., Lan Y.-H., Shi T.-L., Wu D.-X., Hua J. (2019). Natural variations of growth thermo-responsiveness determined by *SAUR26*/*27*/*28* proteins in *Arabidopsis thaliana*. New Phytol..

[30] Qiu T., Qi M., Ding X., Zheng Y., Zhou T., Chen Y., Han N., Zhu M.-Y., Bian H.-W., Wang J.-H. (2020). The *SAUR41* subfamily of small auxin up RNA genes is abscisic acid-inducible to modulate cell expansion and salt tolerance in *Arabidopsis thaliana* seedlings. Ann. Bot..

[31] Jia B., Guo G.-L., Yu T., Dong W.-Y., Zhang S.-Q., Cheng M., Liu L. (2021). Analysis of endogenous IAA content and signaling genes expression in retrieved leaves of ‘Dangshansuli’ Pear (*Pyrus bretschneideri* Rehd.). Acta Bot. Boreali-Occident. Sin..

[32] Mittler R., Suzuki N., Miller G., Tognetti V.-B., Vandepoele K., Gollery M., Shulaev V., Breusegem F.-V. (2011). ROS signaling: The new wave. Trends Plant Sci..

[33] Mittler R., Vanderauwera S., Gollery M., Breusegem F.-V. (2004). Reactive oxygen gene network of plants. Trends Plant Sci..

[34] Verbon E.-H., Trapet P.-L., Stringlis I.-A., Kruijs K., Bakker P.-A.-H.-M., Pieterse C.-M.-J. (2017). Iron and immunity. Annu. Rev. Phytopathol..

[35] Chen W.-W., Yang J.-L., Qin C., Jin C.-W., Mo J.-H., Ye T., Zheng S.-J. (2010). Nitric oxide acts downstream of auxin to trigger root ferric-chelate reductase activity in response to iron deficiency in *Arabidopsis*. Plant Physiol..

[36] Romera F.-J., Garcia M.-J., Alcantara E., Perez-Vicente R. (2011). Latest findings about the interplay of auxin, ethylene and nitric oxide in the regulation of Fe deficiency responses by strategy I plants. Plant Signal. Behav..

[37] Stortenbeker N., Bemer M. (2019). The *SAUR* gene family: The plant’s toolbox for adaptation of growth and development. J. Exp. Bot..

[38] Guo Y., Xu C.-N., Sun X.-J., Hu Z., Fan S.-H., Jiang Q.-Y. (2019). *TaSAUR78* enhances multiple abiotic stress tolerance by regulating the interacting gene *TaVDAC1*. J. Integr. Agric..

[39] He Y.-J., Liu Y., Li M.-Z., Lamin-samu A.-T., Yang D.-D., Yu X.-L., Izhar M., Jan I., Ali M., Lu G. (2021). The *Arabidopsis* small auxin up RNA32 protein regulates ABA-mediated responses to drought stress. Front. Plant Sci..

[40] Zhu X.-F., Wu Q., Meng Y.-T., Tao Y., Shen R.-F. (2020). AtHAP5A regulates iron translocation in iron-deficient *Arabidopsis thaliana*. J. Integr. Plant Biol..

[41] Li D.-Y., Sun Q.-R., Zhang G.-F., Zhai L.-M., Li K.-T., Feng Y. (2021). MxMPK6-2-bHLH104 interaction is involved in reactive oxygen species signaling in response to iron deficiency in apple rootstock. J. Exp. Bot..

[42] Zhu H.-H., Wang J.-Y., Jiang D., Hong Y.-G., Xu J.-M., Zheng S.J., Yang J.-L., Chen W.-W. (2022). The miR157-SPL-CNR module acts upstream of bHLH101 to negatively regulate iron deficiency responses in tomato. J. Integr. Plant Biol..

[43] Bernale M., Monsalve L., Ayala-Raso A., Valdenegro M., Martinez J.-P., Travisany D., Defilippi B., Gonzalez-Aguero M., Cherian S., Fuentes L. (2019). Expression of two indole-3-acetic acid (IAA)-amido synthetase (*GH3*) genes during fruit development of raspberry (*Rubus idaeus* Heritage). Sci. Hortic..

[44] Yue P.-T., Wang Y.-N., Bu H.-N., Li X.-Y., Yuan H., Wang A.-D. (2019). Ethylene promotes IAA reduction through PuERFs-activated *PuGH3.1* during fruit ripening in pear (*Pyrus ussuriensis*). Postharvest Biol. Technol..

[45] Ling H.-Q., Bauer P., Bereczky Z., Keller B., Ganal M. (2002). The tomato fer gene encoding a bHLH protein controls iron-uptake responses in roots. Proc. Natl. Acad. Sci. USA.

[46] Colangelo E.-P., Guerinot M.-L. (2004). The essential basic helix-loop-helix protein FIT1 is required for the iron deficiency response. Plant Cell.

[47] Yuan Y.-X., Zhang J., Wang D.-W., Ling H.-Q. (2005). AtbHLH29 of *Arabidopsis thaliana* is a functional ortholog of tomato FER involved in controlling iron acquisition in strategy I plants. Cell Res..

[48] Wu H., Ling H.-Q. (2019). FIT-binding proteins and their functions in the regulation of Fe homeostasis. Front. Plant Sci..

[49] Cai Y.-R., Yang Y.-J., Ping H.-Q., Lu C.-K., Lei R.-H., Li Y., Liang G. (2022). Why FIT and bHLH Ib interdependently regulate Fe-uptake. Mol. Biol..

[50] Yuan Y., Wu H., Wang N., Li J., Zhao W., Du J., Wang D.-W., Ling H.-Q. (2008). FIT interacts with AtbHLH38 and AtbHLH39 in regulating iron uptake gene expression for iron homeostasis in *Arabidopsis*. Cell Res..

[51] Wang N., Cui Y., Liu Y., Fan H.-J., Du J., Huang Z.-J., Yuan Y.-X., Wu H.-L., Ling H.-Q. (2013). Requirement and functional redundancy of Ib subgroup bHLH proteins for iron deficiency responses and uptake in *Arabidopsis thaliana*. Mol. Plant.

[52] Palmgren M.-G. (2001). Plant plasma membrane H^+^-ATPases: Powerhouses for nutrient uptake. Annu. Rev. Plant Physiol. Plant Mol. Biol..

[53] Henriques R., Jasik J., Klein M., Martinoia E., Feller U., Schell J., Pais M.-S., Koncz K. (2002). Knock-out of *Arabidopsis* metal transporter gene *IRT1* results in iron deficiency accompanied by cell differentiation defects. Plant Mol. Biol..

[54] Bauer P., Ling H.-Q., Guerinot M.-L. (2007). FIT, the FER-like iron deficiency induced transcription factor in *Arabidopsis*. Plant Physiol. Biochem..

[55] Cui Y., Chen C.-L., Cui M., Zhou W.-J., Wu H.-L., Ling H.-Q. (2018). Four IVa bHLH transcription factors are novel interactors of FIT and mediate JA inhibition of iron uptake in *Arabidopsis*. Mol. Plant.

[56] Mittler R. (2017). ROS are good. Trends Plant Sci..

[57] Choudhury F.-K., Rivero R.-M., Blumwald E., Mittler R. (2017). Reactive oxygen species, abiotic stress and stress combination. Plant J..

[58] Marino D., Dunand C., Puppo A., Pauly N. (2012). A burst of plant NADPH oxidases. Trends Plant Sci..

[59] Mark C.-V.-D., Ivanov R., Eutebach M., Maurino V.-G., Bauer P., Brumbarova T. (2021). Reactive oxygen species coordinate the transcriptional responses to iron availability in *Arabidopsis*. J. Exp. Bot..

[60] Song H., Chen F., Wu X., Hu M., Geng Q.-L., Ye M., Zhang C., Jiang L., Cao S.-Q. (2022). *MNB1* gene is involved in regulating the iron-deficiency stress response in *Arabidopsis thaliana*. BMC Plant Biol..

[61] Pence V.-C., Caruso J.-L. (1987). Elisa determination of IAA using antibodies against ring-linked IAA. Phytochemistry.

[62] Bower P.-J., Brown H.-M., Purves W.-K. (1978). Cucumber seedling indoleacetaldehyde oxidase. Plant Physiol..

[63] Koga J., Adachi T., Hidaka H. (1992). Purification and characterization of indolepyruvate decarboxylase. A novel enzyme for indole–3–acetic acid biosynthesis in Enterobacter cloacae. J. Biol. Chem..

[64] Mao F.-C., Zhang F.-Y., Zhao X.-G., Zhang K.-L. (2002). Relativity of active iron contents and chlorosis in the Kiwis leaf. Acta Agric. Boreali-Occident. Sin..

[65] Chen W.-W., Jin J.F., Lou H.-Q., Liu L., Kochian L.-V., Yang J.-L. (2018). LeSPL–CNR negatively regulates Cd acquisition through repressing nitrate reductase-mediated nitric oxide production in tomato. Planta.

[66] Aksoy E., Koiwa H. (2013). Determination of ferric chelate reductase activity in the Arabidopsis thaliana root. Bio-Protocol.

[67] Schmidt W. (2003). Iron solutions: Acquisition strategies and signaling pathways in plants. Trends Plant Sci..

[68] Aono M., Kubo A., Saji H., Tanaka K., Kondo N. (1993). Enhanced tolerance to photooxidative stress of transgenic *Nicotiana tabacum* with high chloroplastic glutathione reductase activity. Plant Cell Physiol..

[69] Kumar D., Yusuf M.-A., Singh P., Sardar M., Sari N.-B. (2014). Histochemical detection of superoxide and H_2_O_2_ accumulation in *Brassica juncea* seedlings. Bio-Protocol.

[70] Lin C.-C., Kao C.-H. (2001). Cell wall peroxidase activity, hydrogen peroxidase level and NaCl-inhibited root growth of rice seedling. Plant Soil.

[71] Bai S., Tao R., Tang Y., Yin L., Ma Y.-J., Ni J.-B., Yan X.-H., Yang Q.-S., Wu Z.-Y., Zeng Y.-L. (2019). BBX16, a B-box protein, positively regulates light-induced anthocyanin accumulation by activating MYB10 in red pear. Plant Biotechol. J..

[72] Fillatti J.-J., Kiser J., Rose R., Comai L. (1987). Efficient transfer of a glyphosate tolerance gene into tomato using a binary *agrobacterium* tumefaciens vector. Nat. Biotechol..

[73] Clough S.-J., Bent A.-F. (1998). Floral dip: A simplified method for *agrobacterium*-mediated transformation of *Arabidopsis thaliana*. Plant J..

[74] Livak K.-J., Schmittgen T.-D. (2002). Analysis of relative gene expression data using real-time quantitative PCR. Methods.

