# Supplementary material for: Evidence That PbrSAUR72 Contributes to Iron Deficiency Tolerance in Pears by Facilitating Iron Absorption
Source: Plants (Basel). 2023 May 30;12(11):2173. doi: 10.3390/plants12112173 (PMC10255822; doi:10.3390/plants12112173)
Supplement: Supplementary file 1 [file plants-12-02173-s001.zip › plants-2334887-SI.pdf]

plants

Article

Evidence That *PbrSAUR72* Contributes to Iron Deficiency Tolerance in Pears by Facilitating Iron Absorption

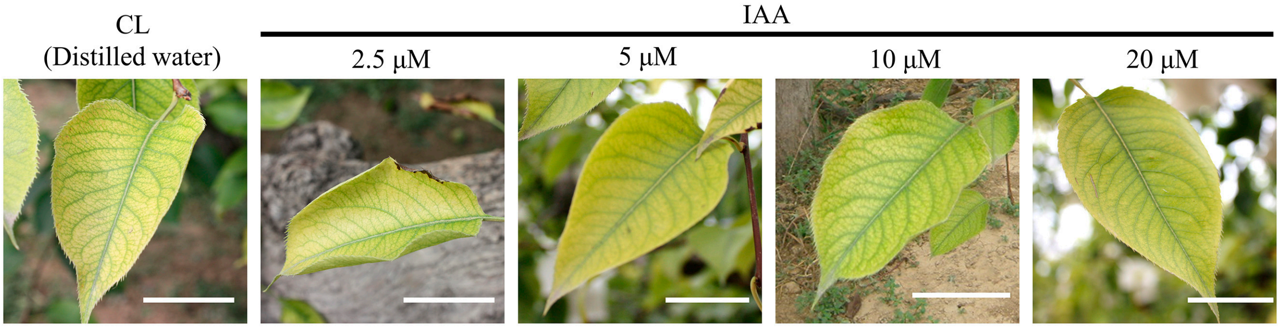

Figure S1. Representative images of CL treated with different doses of indoleacetic acid (IAA). IAA of 2.5, 5, 10, and 20 μM concentrations were sprayed evenly on CL, and distilled water used as the control. The color changes of CL were recorded at 14 d after treatments. Bars = 5 cm.

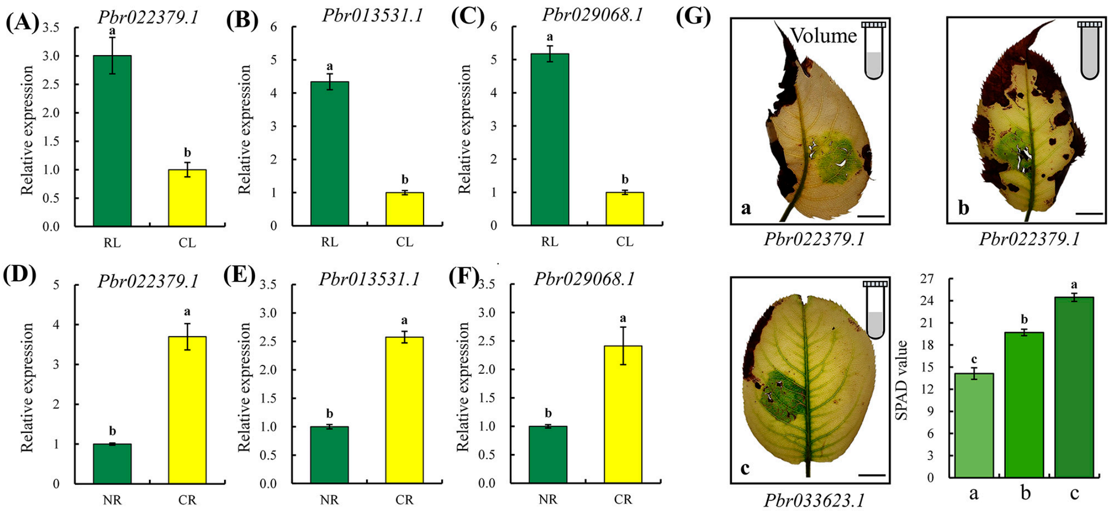

Figure S2. *PbrSAUR* expression analysis in leaf and roots of pear trees. The relative *PbrSAUR* expression levels in regreening leaf (RL), chlorotic leaf (CL), and the roots of normal pear trees (NR) and chlorotic pear trees (CR) were verified by qRT−PCR, including *Pbr023379.1*, *Pbr013531.1*, and *Pbr029068.1*. *PbrGAPDH* was used as the internal control. (G) The gene expression level was not the decisive factor of the regreening degree. Suspensions with different dosages of *Pbr033623.1* (*PbrSAUR72*) and *Pbr022379.1* were injected into the CL via syringes, and the phenotypes were observed at 14 d after injection. The gray part of the test tube in the upper right corner of the images was the dosage used. Bars = 1 cm.

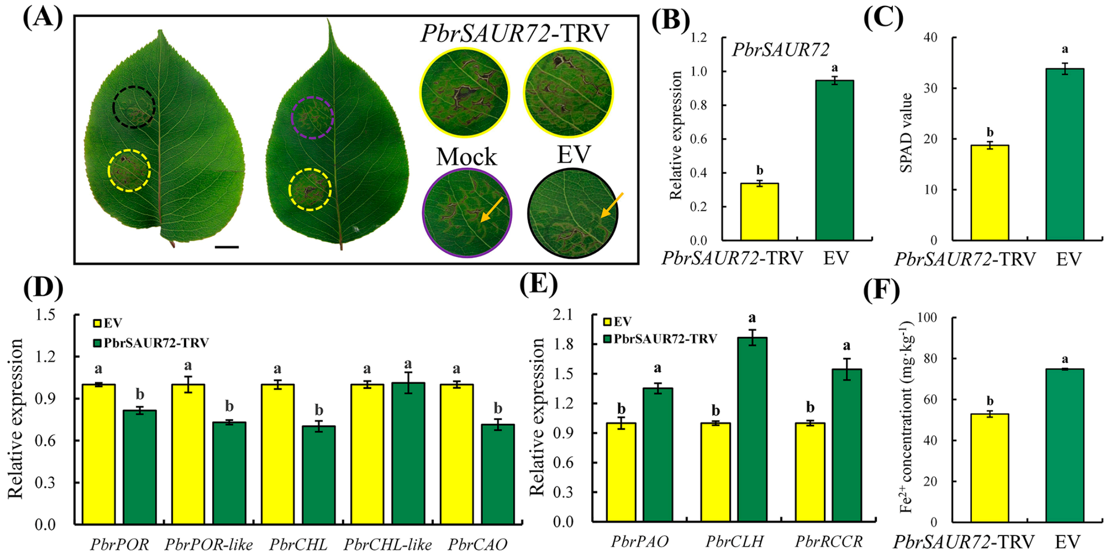

Figure S3. Transiently silencing *PbrSAUR72* aggravates chlorophyll degradation and receding Fe^{2+} accumulation in normal leaf of pear (*Pyrus bretschneideri* Rehd.). (A) The function of PbrSAUR72 was silenced in NL via virus−induced gene silencing method. A mixture containing equal volumes of empty vector (EV) and pTRV1 was used as a control. The phenotypes were observed at 14 d after injection. Meanwhile, (B) the corresponding genes' expression and (C) chlorophyll content in the injection site were tested to show leaf color alteration. (D) Expression of genes related to chlorophyll synthesis and (E) chlorophyll degradation was calculated by qRT−PCR. The expression level detected in EV was used as the reference and set to '1'. *PbrGAPDH* was used as the internal control. (F) Analysis of Fe^{2+} content in the injected spot. Values shown are the mean ± SE (n = 3) of three biological replicates. Student's *t*−test: Different lowercase letters above the columns indicate a significant difference at $p < 0.05$.

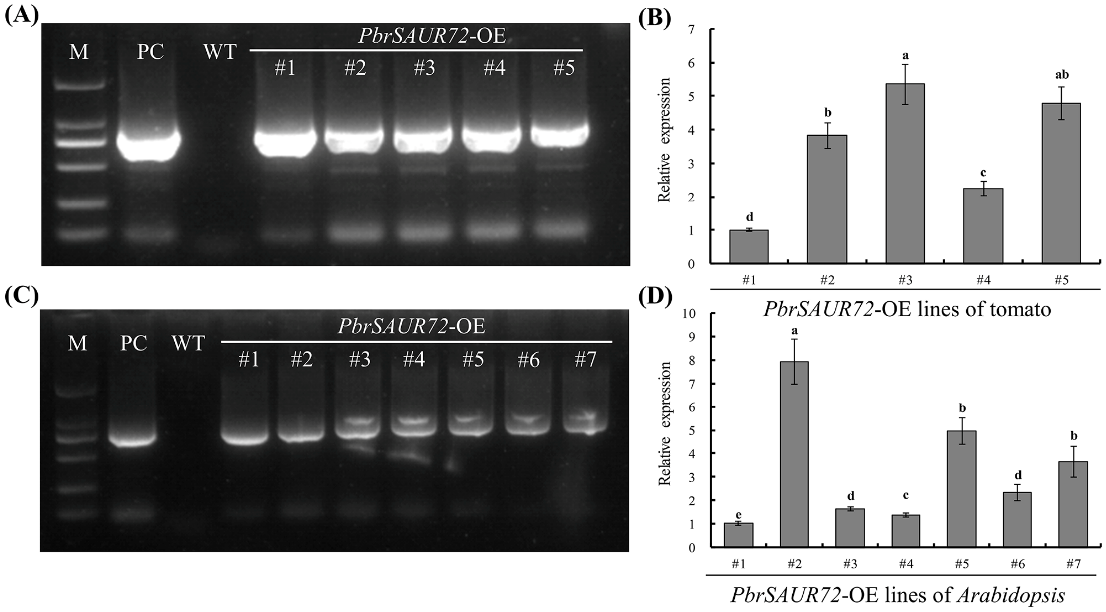

Figure S4. Molecular identification of *PbrSAUR72*–OE transgenic plants. Detection of *PbrSAUR72*–OE tomato (A) and *Arabidopsis* plants (C) at the DNA level. M, DNA Marker 2000; PC, positive control with *PbrSAUR72*–Flag as a template; WT, negative control with gDNA from *Arabidopsis thaliana* and *Solanum lycopersicum*, respectively. A sequence from the rice Ubi1 promoter of p1300U1–Flag vector was used as the forward primer (ATTTTGTTAGGTCACTGTTGGTGT) and a sequence from p1300U1–Flag vector after the insertion sites (AGCTACTTGTCATCGTCATCCTTG) was used as the reverse primer. Transcript activities of *PbrSAUR72* in the transgenic lines of tomato (B) and *Arabidopsis* (D), as determined by qRT–PCR. The lowest expression detected in transgenic plants served as the reference and was set to '1'. *SlActin7* and *AtActin2* were used as the internal controls. Data are the mean ± SE (n = 3) of three biological replicates. student's *t*–test: Different lowercase letters above the columns indicate a significant difference at $p < 0.05$.

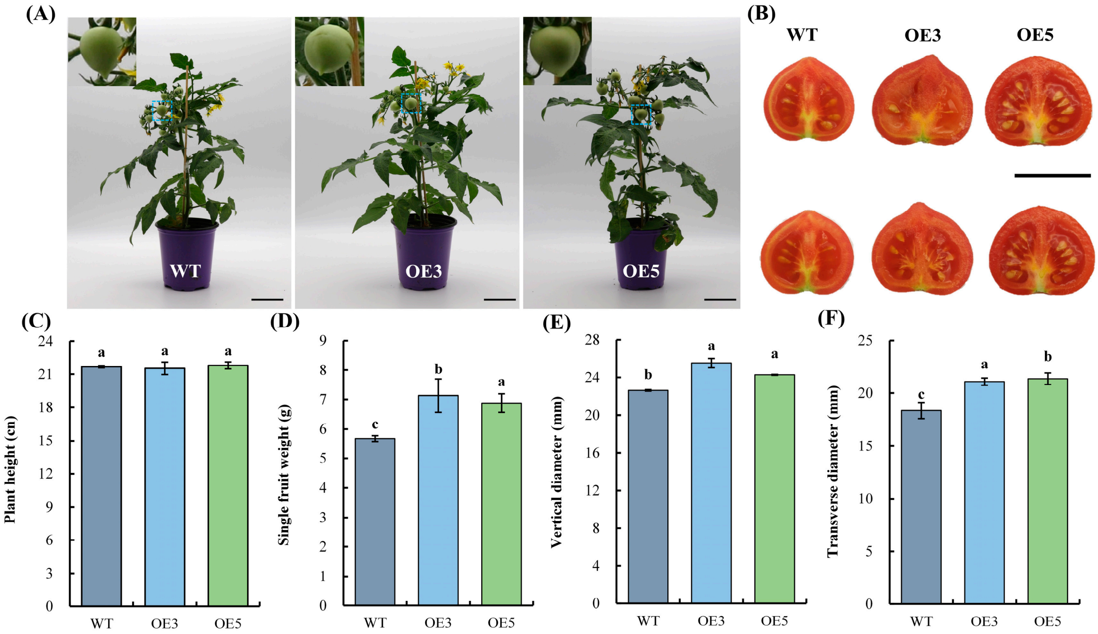

Figure S5 Representative images of whole plants and fruits for transgenic and wild type (WT) tomato plants. (A) Phenotypes of approximately 90–day–old transgenic and WT tomato plants, and fruits at the mature green stage were presented in a partially enlarged form. Scale bare = 5 cm. (B) The cross–section of transgenic and WT tomato fruits at the mature red stage. Scale bar=2 cm. (C) The height of approximately 90–day–old transgenic and WT tomato plants. (D) Single fruit weight, (E) vertical diameter, and (F) transverse diameter of transgenic and WT tomato fruits at the mature red stage.

Table S1. The list of primers used in this study for qRT–PCR and vectors construction.

Gene ID	Name	Primer Sequences (5'→3')	Gene ID	Name	Primer Sequences (5'→3')
**For qRT-PCR analysis (Fe-related)**					
Pbr033623.1	*PbrSAUR72*	F–GGGGTGTTGTTGTAAAGGGAAG<br>R–GAAACAGAACACAGAATTTGGGAC	*Solyc10g079680*	*SlbHLH101*	F-TTGTTTGAAGGTTTACGTGCCG<br>R–AGTAGGAGGAGGAAGCAAAGAAC
Pbr022379.1,	*PbrSAUR50*	F–CCCATCATCGCCATTACCTC<br>R–AACCGCTTGTAGCTGCTTCC	*Solyc08g081690*	*SlRBOH1*	F-TGGGGATGACTACTTGAGCA<br>R-AAGCCTCGGAAAACACTCG
Pbr013531.1	*PbrSAUR32*	F–GGTTTGTTATTCCGGTGGTCT<br>R–GATGGTGGTGGTTGTGATGGT	*AT3G18780*	*AtActin2*	F–GGTAACATTGTGCTCAGTGGTGG<br>R–AACGACCTTAATCTTCATGCTGC
Pbr029068.1	*PbrSAUR78*	F–GTGGCTTGTGAGGTGGTGCT<br>R–CACACATAGCAGCAGCCAGC	*AT2g28160*	*AtFIT*	F–GGAGAAGGTGTTGCTCCATC<br>R–TCCGGAGAAGGAGAGCTTAG
Pbr024344.1	*PbrGAPDH*	F–TGGTGTCATGGTTGGTATGG<br>R–CAGGAGCAACACGAAGTTCA	*AT4g19690*	*AtIRT1*	F–AAGCTTTGATCACGGTTGG<br>R–TTAGGTCCCATGAACTCCG
Solyc03g078400	*SlActin7*	F– CAGCAGATGTGGATCTCAAA<br>R– CTGTGGACAATGGAAGGAC	*AT1g01580*	*AtFRO2*	F–CTTGGTCATCTCCGTGAGC<br>R–AAGATGTTGGAGATGGACGG
Solyc01g094910	*SlFRO1*	F–GCTCGTGTTCTGCCATGTGA<br>R–GCAACACTGATTTTCTCTGGCT	*AT4G30190*	*AtAHA2*	F–GAGATGCGTGTTCCACACGTTT<br>R–AGAGATGGCAGGAATGGATG
Solyc02g069200	*SlIRT1*	F–ATTCCTACTCAAAATTCAAA<br>R–ACAAACTCCGATCATACTAG	*AT3g56970*	*AtbHLH38*	F–AGCAGCAACCAAAGGCG<br>R–CCACTTGAAGATGCAAAGTGTAG
Solyc12g010360.2	*SlHA4*	F–TATCAGAGCCTGCCATTCCCT<br>R–AACCACCAGAAGCATCAAACC	*AT3g56980*	*AtbHLH39*	F–GACGGTTTCTCGAAGCTTG<br>R–GGTGGCTGCTTAACGTAACAT
Solyc06g051550.3	*SlFER*	F–TGAATCTTCTGGCACAACG<br>R–CCAATGATGGAGGCTTTATC	*AT2g41240*	*AtbHLH100*	F–AAGTCAGAGGAAGGGGTTACA<br>R–GATGCATAGAGTAAAAGAGTCGCT
Solyc10g079650	*SlbHLH39*	F–CCCATGGAACCTCTTCATTCATC<br>R–GTTGATGATGATCACCACCGTTG	*AT5g04150*	*AtbHLH101*	F–CAGCTGAGAAACAAAGCAATG<br>R–CAGTCTCACTTTGCAATCTCC
Solyc10g079660	*SlbHLH100*	F–AGGCAAGCTGCTGAGACATT<br>R–CCAAGTCACCCCTGACATCT			
**For qRT–PCR analysis (Chlorophyll synthesis–related)**					
Pbr022321.1	*PbrPOR*	F–AGTGTTGGGACTAACCATCTCG<br>R–GCACCGTCAAAGTCTCCACC	*Pbr028856.1*	*PbrCHL*	F–GGCAGGACATAACTTCCCAATA<br>R–AATTCCCAACCCAGCTATGC
Pbr036353.1	*PbrPOR-like*	F–GGGGAAGAAGACTCTAAGAAAGG<br>R–ACGCCTGAAGTTATCAACGAAT	*Pbr019452.1*	*PbrCHL-like*	F–TTATTTGGGACCCTTACGCC<br>R–GCCCCTAGCAGATAACCAGC
Pbr002838.1	*PbrCAO*	F–CTCTTCCTTCTTTACAACCTCCTG<br>R–ACAAGGTGGTCGAAATTCCAT	*Pbr033572.2*	*PbrPAO*	F–TGCTGATTTATTTGATGGCTCTG<br>R–CATGATGCTCCCATATCGACTG
Pbr012920.1	*PbrPCLH*	F–TCTTGCTTCATCACGGCTTCT<br>R–AGGTCCACTTGGAGGCACA	*Pbr030421.1*	*PbrRCCR*	F–AGAGCAGCCCAACATCCCTA<br>R–CCACTCCCTGCTTCGATCC
**For the *PbrSAURs* overexpression and GFP vectors generation**					
Pbr033623.1	*PbrSAUR72*–Flag	F–TGTGCAGCCCGGGATCCATGAAGCAGCTGATCCGCC<br>R–CAATTGACGCGTGTCGACCCAGGATCTCAGGTCGACG		*PbrSAUR72*–GFP	F–AACACGGGGGACTCTAGAATGAAGCAGCTGATCCGCC<br>R–CCTTGCTCACCATGGATCCCCAGGATCTCAGGTCGACG
Pbr033623.1	*PbrSAUR72*–TRV	F–AAGGTTACCGAATTCATGAAGCAGCTGATCCGCC<br>R–GCTCGGTACCGGATCCCCAGGATCTCAGGTCGACGG	*Pbr013531.1*	*PbrSAUR32*–Flag	F–TGTGCAGCCCGGGATCCATGGGATTTATCGCCGCCGA<br>R–CAATTGACGCGTGTCGACAACCCTAAAACACCAGGC
Pbr022379.1	*PbrSAUR50*–Flag	F–TGTGCAGCCCGGGATCCATGTCGCCGGTGATCAGC<br>R–CAATTGACGCGTGTCGACGCCAACCCGAGCGCG	*Pbr029068.1*	*PbrSAUR78*–Flag	F–TGTGCAGCCCGGGATCCATGGCAAAAGCCGGGAAGCT<br>R–CAATTGACGCGTGTCGACGCAAGTGTAGAACTCAA
**For transgenic plant identification**					
	JC	F–ATTTTGTTAGGTCACTGTTGGTGT<br>R–AGCTACTTGTCATCGTCATCCTTG			

Note: Bold and underline letters represent restriction enzyme sites.

Table S2. Transcriptome analysis of differentially expressed genes (DEGs) of SAUR. in regreening leaf (RL) and chlorotic leaf (CL) of pear.

ID	Expression (FPKM)		ID	Expression (FPKM)		ID	Expression (FPKM)	
	CL	RL		CL	RL		CL	RL
Pbr001267.1	0.22	0.37	*Pbr026939.1*	0.79	0.42	*Pbr028075.1*	0.99	8.57*
Pbr033623.1	**6.46**	**35.05***	*Pbr009926.1*	0.66	0.94	*Pbr005852.1*	3.18	1.55*
Pbr023255.1	2.21	7.80*	***Pbr013531.1***	**19.12**	**103.25***	*Pbr018494.1*	128.03	123.62
Pbr020542.1	25.99	26.51	*Pbr035340.1*	7.37	2.35*	*Pbr042229.1*	17.93	1.70*
Pbr004491.1	0.64	0.81	*Pbr018755.1*	2.13	1.10	*Pbr041090.1*	33.74	40.93*
Pbr041058.1	0.97	1.49*	*Pbr024944.1*	0.38	4.85*	*Pbr028073.1*	1.32	0.28*
Pbr003402.1	0.50	0.73	*Pbr042213.1*	0.87	1.54*	*Pbr041217.1*	0.51	0.40
Pbr018404.1	3.83	1.53	*Pbr014730.1*	0.32	0.52	*Pbr007826.1*	5.40	0.51*
Pbr022379.1	**8.28**	**64.88***	***Pbr029068.1***	**11.39**	**40.36***	*Pbr042214.1*	0.33	0.19
Pbr001262.1	0.22	0.37	*Pbr020564.1*	0.38	4.85*	*Pbr009936.1*	2.32	0.69*
Pbr036005.1	0.25	0.54	*Pbr040072.1*	42.83	56.52	*Pbr037398.1*	0.22	0.35

Note: '*' means a significant difference between RL and CL, and bold font represent significantly expressed genes in RL with high expression (FPKM≥10).
